# Assessment of Knowledge, Attitudes, and Preventive Practices for Equine Endoparasite Control Among Livestock Owners in South Gondar Zone, Northwest Ethiopia

**DOI:** 10.1155/vmi/5535645

**Published:** 2026-07-22

**Authors:** Habtamu Alemayehu, Tewodros Alemneh, Zewdu Seyoum, Moges Maru, Abrham Ayele, Mastewal Birhan

**Affiliations:** ^1^ Fogera District Livestock and Fishery Resources Development Office, South Gondar Zone, Woreta, Ethiopia; ^2^ Department of Veterinary Pathobiology, College of Veterinary Medicine and Animal Sciences, University of Gondar, P.O. Box 196, Gondar, Ethiopia, uog.edu.et; ^3^ Woreta Town Agriculture, and Livestock and Fishery Resources Development Office, South Gondar Zone, Woreta, Ethiopia

**Keywords:** endoparasites, equine, Ethiopia, knowledge-attitude-practice, logistic regression

## Abstract

Endoparasites are a major health concern affecting equine productivity and welfare worldwide. Understanding farmers’ knowledge, attitudes, and practices (KAP) toward parasite control is critical for designing effective interventions. This study assessed KAP levels among farmers in South Gondar Zone, Northwest Ethiopia, and identified factors associated with good KAP. A community‐based cross‐sectional study was conducted among 150 farmers. KAP scores were initially categorized into three levels (low, moderate, and high) and analyzed using chi‐square (*χ* ^2^) tests to explore bivariate associations. For logistic regression analyses, KAP outcomes were dichotomized into good and poor categories. Univariable and multivariable binary logistic regression models were used to identify independent predictors of good KAP, with *p* ≤ 0.05 considered statistically significant. The study employed representative sampling across districts, a pretested questionnaire, and comprehensive adjustment using multivariable logistic regression to enhance the reliability of the findings. Among 150 respondents, 120 (80%) were male and 30 (20%) female. The majority were adults (50.7%), followed by young (34.7%) and old (14.7%) age groups. Nearly half of the participants were with no formal education (49.3%), 44% had primary education, and 6.7% had secondary education. Farmers were evenly distributed across three districts (Dera, Fogera, and Libokemkem), and most (*n* = 123, 82%) lived within 1–10 km of a veterinary facility. Overall, 52.7% of farmers demonstrated good knowledge, 66.7% exhibited a positive attitude, and 91.3% reported good practices toward equine endoparasites. Multivariable binary logistic regression analysis using SPSS (v. 27) revealed that, for knowledge, adults (AOR = 0.12, 95% CI: 0.03–0.50) and older farmers (AOR = 0.10, 95% CI: 0.02–0.55) were less likely to have good knowledge compared to young farmers, whereas educated farmers were substantially more likely to have good knowledge (AOR = 15.8, 95% CI: 3.4–73.8). Regarding attitude, adults (AOR = 0.015, 95% CI: 0.002–0.12) and older farmers (AOR = 0.013, 95% CI: 0.001–0.12) were less likely to have a positive attitude, while education (AOR = 18.5, 95% CI: 5.0–68.0) and residence in Libokemkem district (AOR = 3.80, 95% CI: 1.36–10.57) were positively associated with good attitude. For practice, good practices were more likely among adult farmers (AOR = 54.5, 95% CI: 4.8–620), but less likely among females (AOR = 0.18, 95% CI: 0.04–0.78) and farmers living > 10 km from veterinary services (AOR = 0.06, 95% CI: 0.004–0.95). Age and education were key determinants of farmers’ KAP toward equine endoparasite control. Hence, targeted educational interventions focusing on different age and education groups could improve parasite management practices in this region.

## 1. Introduction

Livestock farming is a cornerstone of Ethiopia’s agricultural economy and a critical source of livelihood, food security, and draught power for millions [1]. Within this sector, equids (horses; donkeys, and mules) play an indispensable role. They are vital for rural transport, plowing, and threshing, directly contributing to agricultural productivity and household income, particularly in the mixed crop–livestock systems common in the Amhara Region [2, 3]. However, the health and productivity of these essential animals are persistently undermined by gastrointestinal parasites, which rank among the most common and debilitating health challenges in equines worldwide [4]. Infections can lead to clinical disease and poor body condition, which in turn reduces work capacity, and can also increase mortality, resulting in significant economic losses for the owners who depend on them [5]. Among the various parasites affecting equids, strongyles, ascarids, and tapeworms are particularly significant due to their high prevalence and substantial impacts on the health and welfare of working equines [6, 7].

Effective control of these endoparasites requires an integrated strategy. Contemporary veterinary parasitology emphasizes sustainable practices that combine strategic, evidence‐based anthelmintic (dewormer) use with fundamental husbandry measures such as regular manure removal, pasture rotation, and proper sanitation [8]. The success of any such control program, however, is ultimately dependent not just on the availability of drugs or guidelines, but on the knowledge, attitudes, and practices (KAP) of the animal owners. The KAP framework is a well‐established tool in public and veterinary health used to understand what people know (knowledge), how they feel (attitude), and what they do (practice) regarding a health issue [9]. This understanding is crucial because even the best technical recommendations fail if they are not adopted or are incorrectly implemented by the end‐user [10, 11].

Recent studies in Ethiopia have begun to illuminate the human‐behavioral dimension of animal health. Research by Yizengaw et al. [3] in the East Gojjam Zone of the Amhara Region revealed that while livestock owners possessed relatively good knowledge (57%) and positive attitudes (69%) toward disease prevention, their actual reported practices were notably poor (49%). This pervasive “know‐do” gap, where awareness does not translate into action, has been attributed to barriers such as limited access to veterinary services, economic constraints, and educational levels. Similar gaps between knowledge and practice in parasite control have been documented among livestock farmers in other settings, such as Iran [12]. Importantly, interventions based on KAP assessments have proven effective; for instance, targeted education has been shown to significantly improve knowledge of equine parasitic diseases among caretakers [13].

Despite these advances, a significant research gap remains. While broad livestock KAP studies like that of Yizengaw et al. [3] provide essential regional context, there is a notable lack of targeted research focusing specifically on equine endoparasite control in the South Gondar Zone. Existing studies on equine health in Ethiopia often concentrate on clinical prevalence (e.g., Ref. [7]) or anthelmintic efficacy (e.g., Ref. [14]), leaving the underlying drivers of owner behavior, namely, their specific knowledge, attitudes toward responsibility and risk, and routine management practices, largely unexplored. To the authors’ knowledge, this is the first equine‐specific KAP study conducted, addressing a critical evidence gap for the development of targeted interventions aimed at improving equine parasite control, welfare, and productivity in the region. This specific understanding is a prerequisite for designing effective, culturally appropriate, and sustainable extension programs to improve equine welfare and productivity in the zone.

Therefore, this community‐based study aimed to assess the KAP of farmers regarding equine endoparasite control in the South Gondar Zone, Northwest Ethiopia. Specifically, it sought first to evaluate the level of farmer knowledge concerning parasite transmission, clinical signs, and health impacts. Second, it aimed to determine their attitudes toward the seriousness of infections and their perceived responsibility for control. Third, the study documented the prevailing preventive and control practices, including deworming strategies and stable hygiene. Finally, it identified key sociodemographic and management factors associated with adequate knowledge and desirable practices to inform future interventions.

## 2. Materials and Methods

### 2.1. Study Area

**FIGURE 1:**
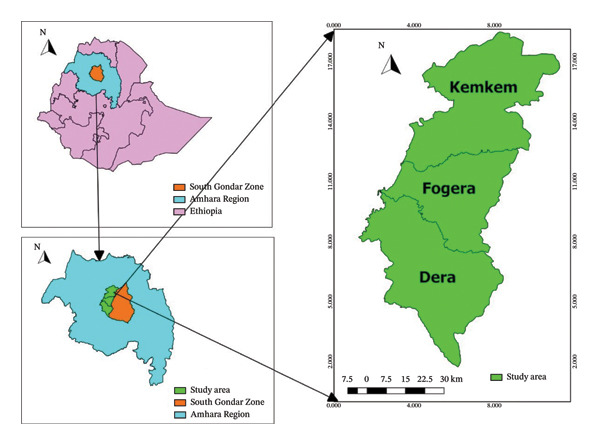
Map of the study area (source: authors, 2026 using QGIS Version 3.32).

The study was conducted in South Gondar Zone, a governmental administrative zone of the Amhara Region in Ethiopia, with Debre Tabor town as its capital, located 666 km from Addis Ababa and 103 km from Bahir Dar [15]. The zone lies between 11°02′‐12°33′ N latitude and 37°25′‐38°43′ E longitude and covers an area of approximately 14,320 km^2^ [16]. Administratively, it consists of 18 districts (13 rural and 5 urban) and 405 kebeles (364 rural and 41 urban) and is bordered to the south by East Gojjam, to the west by Lake Tana, to the north by North Gondar, and to the east by North Wollo [15]. The zone is characterized by diverse topography, ranging from flat and low‐lying grazing lands to high cold mountainous areas and is agroecologically classified into highland (“dega”) and mid‐altitude (“woina dega”) zones. Elevation ranges from approximately 1500 to 3600 m above sea level (m.a.s.l) [17]. Climatically, the zone experiences annual minimum and maximum temperatures of about 17°C and 27°C, respectively. Rainfall distribution is predominantly monomodal, extending from June to mid‐September, with peak rainfall occurring between July and August, while mean annual rainfall varies widely from 500 mm to 1600 mm [18]. South Gondar Zone is well known for livestock production, particularly for its indigenous cattle breeds such as the “Fogera” cattle, and dairying is commonly practiced using small herd sizes [17]. According to the Central Statistical Agency [19], the zone has an estimated livestock population of 1,977,764 cattle, 1,225,461 sheep, 905,804 goats, 34,417 horses, 24,723 mules, and 406,620 donkeys. Map of the study area is shown in Figure 1.

### 2.2. Study Design and Period

A community‐based cross‐sectional study design was employed to assess the KAP of farmers regarding equine endoparasites management and control. The data collection was conducted over an 11‐month period, from September 2023 to July 2024.

### 2.3. Study Population

The source population comprised all households owning equines (horses, mules, and donkeys) in the selected districts of the study area. The study population consisted of adult household members aged 18 years or older who were primarily responsible for the daily care, management, and health‐related decision‐making of equines within their households. Only one eligible respondent was interviewed per household to the avoid duplication of information.

Sociodemographic and farm‐related characteristics of respondents were collected and categorized as follows: Gender was classified as male or female. Age of respondents was grouped into three categories: less than 30 years (young), 30–45 years (adult), and greater than 45 years (older), consistent with classifications commonly used in social science and livestock‐related studies [3, 20, 21]. Educational status was categorized into four levels: illiterate (no formal education), primary education, secondary education, and higher education. Access to veterinary services was assessed based on the reported distance from the respondents’ residence to the nearest veterinary facility and categorized into four groups: < 1 km, 1–5 km, 5–10 km, and > 10 km. Primary source of income was categorized as crop production, livestock rearing, or mixed farming. Farmers with more than 1 year of experience raising equids were included. Administrative location was classified by district, namely, Dera, Fogera, and Libokemkem. These classifications were used consistently throughout data analysis to assess the variations in KAP related to equine endoparasites among respondents.

### 2.4. Sample Size Determined and Sampling Technique

The sample size was determined using the formula for estimating a single population proportion with a specified absolute precision according to Taherdoost [22] as follows:

(1) n=0.25SE2,

where SE is the standard error.

Because no prior data were available on the KAP of farmers regarding equine endoparasites control in the study area, a conservative proportion of 0.25 was used to maximize sample size. A standard error of 0.0408 was selected, corresponding to a margin of error of 8% at the 95% confidence level. This yielded a minimum required sample size of 150 farmers.

**FIGURE 2:**
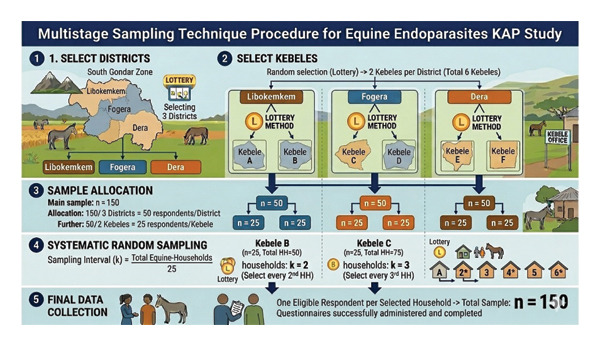
Framework of multistage sampling for selecting the study population.

A multistage sampling technique was employed (Figure 2). First, the South Gondar Zone was purposively selected from the Amhara National Regional State based on its high equine population and accessibility for the study. Second, three districts (Fogera, Dera, and Libokemkem) were randomly selected from South Gondar Zone. Third, from each district, two kebeles (the smallest administrative units) were selected randomly using the lottery method. The total sample size (*n* = 150) was equally allocated among the three selected districts (50 respondents per district) and further distributed equally to the two kebeles within each district, resulting in 25 respondents per kebele.

Finally, from lists of equine‐owning households obtained from kebele Livestock and Fishery Resources Development Offices, the required number of households per kebele was selected using systematic random sampling. The sampling interval (*k*) was determined by dividing the total number of equine‐owning households in each kebele by the allocated sample size (i.e., 25). Because the total number of equine‐owning households per kebele ranged from 50 to 75, the sampling intervals were either 2 (for 50 households) or 3 (for 75 households). The first household was then selected randomly (by the lottery method), and subsequent households were selected at regular intervals (every k^th^ household) until the required sample size was achieved. From each selected household, one eligible respondent (aged ≥ 18 years and involved in equine management or healthcare decision‐making) was interviewed. When more than one eligible respondent was available in a household, one respondent was selected using the lottery method. A total of 150 questionnaires were successfully administered and completed.

### 2.5. Data Collection Tool and Procedure

Data were collected using a structured, pretested, and interviewer‐administered questionnaire (Appendix 1). It comprised four main sections: first, sociodemographic and farm characteristics, including gender, age, education level, access to veterinary facilities, and grazing system; second, knowledge assessment, with eleven (11) items exploring causes, transmission routes, clinical signs, and health impacts of equine endoparasites (responses: Yes, No, I don’t know); third, attitude assessment, consisting of seven items (A1–A7) measured on a three‐point scale (Agree, Neutral, Disagree) to gauge perceptions of severity, responsibility, and consequences; and fourth, practice assessment, with seven items (P1–P7) documenting deworming frequency, decision‐making, and biosecurity (husbandry) practices related to endoparasite management and control. The questionnaire was pretested on 15 farmers (five from each district) outside the final study kebeles to check clarity, flow, and time requirement and was refined accordingly. Six trained diploma‐level animal health technicians conducted the face‐to‐face interviews at respondents’ homes.

### 2.6. Operational Definitions and Data Management

For analysis, composite scores were created for each KAP domain. Knowledge was assessed by assigning 1 point for each correct answer and 0 points for incorrect or “I don’t know” responses, and the total score (out of 11) was converted to a percentage. Initially, based on methodologies used in similar KAP studies [23–25], scores were categorized as High (75%–100%), Moderate (51%–74%), and Low (0%–50%). Attitude was measured by assigning 2 points for “Agree”, 1 points for “Neutral”, and 0 point for “Disagree” for positively framed statements, while negatively framed statements were reverse‐scored; the total score was then converted to a percentage and categorized using the same thresholds as Good (75%–100%), Moderate (51%–74%), and Poor (0%–50%). Practice was evaluated by assigning 1 point for each reported adherence to a recommended practice and 0 otherwise. The total score (out of 7) was converted to a percentage and categorized as Good (75%–100%), Moderate (51%–74%), or Poor (0%–50%).

Subsequently, for univariable and multivariable binary logistic regression analyses, KAP outcomes were dichotomized into Poor (Low) and Good (Moderate and High combined) categories to ensure adequate sample size per group and improve model stability. Collected data were checked daily for completeness and then coded and entered into Microsoft Excel 2010; after comprehensive cleaning, the data were prepared for statistical analysis.

### 2.7. Statistical Analysis

Descriptive statistics, including frequencies, percentages, and means, were used to summarize the sociodemographic and farm‐related characteristics of the respondents and the distribution of responses in the KAP sections. Associations between independent variables (gender, age, education level, access to veterinary services, income source, and district) and the primary binary outcomes (good knowledge, favorable attitude, and good practice) were initially examined using bivariate analyses. The chi‐square (*χ* ^2^) test of independence was applied when expected cell frequencies were adequate. When more than 20% of the cells had expected counts less than five, exact tests were employed. Specifically, for contingency tables larger than 2 × 2, the Fisher–Freeman–Halton (FFH) exact test (an extension of Fisher’s exact test designed for *R* × *C* tables) was used, as Fisher’s exact test is restricted to 2 × 2 tables. Variables with a *p* value of ≤ 0.05 in the bivariate analyses were considered statistically significant at this stage.

To control for potential confounding and identify independent predictors of KAP outcomes, univariable binary logistic regression analyses were first performed for each independent variable. Variables with a *p* value of < 0.25 in univariable binary logistic regression analyses were considered candidates for the multivariable model, in accordance with recommended model‐building strategies [26, 27]. A multivariable binary logistic regression model was then fitted using a backward stepwise elimination procedure. Variables with a *p* value of ≤ 0.05 were retained in the final model. The strength of associations is presented as adjusted odds ratios (AORs) with corresponding 95% confidence intervals (CIs). All statistical analyses were performed using IBM SPSS Statistics, Version 27.

## 3. Results

### 3.1. Socio‐Demographic and Spatial Characteristics of Respondents

A total of 150 livestock farmers were included in the study to assess their KAP regarding equine endoparasites. The majority of respondents were males (80.0%, *n* = 120). Adult farmers constituted the largest age group (50.7%, *n* = 76), followed by young (34.7%, *n* = 52) and older farmers (14.7%, *n* = 22). Nearly half of the respondents were illiterate (49.3%, *n* = 74), while 44.0% (*n* = 66) had completed primary education, and 6.7% (*n* = 10) had secondary education; none of the respondents reported higher education. Spatial analysis revealed that most farmers lived 1–10 km from veterinary facilities (76.7%, *n* = 115), with only 5.3% (*n* = 8) residing within 1 km and 18.0% (*n* = 27) beyond 10 km. Participants were evenly distributed across the three study districts (Dera, Fogera, and Libokemkem), each representing 33.3% (*n* = 50) of the sample. The complete distribution of sociodemographic and spatial characteristics is presented in Table 1.

**TABLE 1:** Sociodemographic and spatial characteristics of farmers participating in a KAP assessment on equine endoparasites (n = 150).

Variable	Category	Frequency (*n*)	Percentage (%)	95% CI (%)
Gender	Male	120	80.0	73.6–86.4
	Female	30	20.0	13.6–26.4

Age	Young	52	34.7	27.1–42.3
	Adult	76	50.7	42.7–58.7
	Old	22	14.7	9.0–20.3

Education	Illiterate	74	49.3	41.3–57.3
	Primary	66	44.0	36.1–51.9
	Secondary	10	6.7	2.7–10.7
	Higher	0	0.0	—

Income source	Livestock rearing	0	0.0	—
	Crop production	0	0.0	—
	Mixed farming	150	100	—

Vet facility distance	< 1 km	8	5.3	1.7–8.9
	1–5 km	56	37.3	29.6–45.1
	5–10 km	59	39.3	31.5–47.2
	> 10 km	27	18.0	11.9–24.1

District	Dera	50	33.3	25.8–40.9
	Fogera	50	33.3	25.8–40.9
	Libokemkem	50	33.3	25.8–40.9

Total		150	100	—

### 3.2. Knowledge of Equine Endoparasites Among Livestock Farmers

Respondents were assessed for their knowledge of equine endoparasites. Overall, respondents demonstrated a generally high to moderate level of knowledge, with 52.7% (*n* = 79) classified as having high knowledge and 47.3% (*n* = 71) as having moderate knowledge. No respondents were classified in the low knowledge category.

Chi‐square and FFH exact tests indicated statistically significant associations between the knowledge level and age as well as educational status. Age was significantly associated with knowledge level (*χ* ^2^ = 65.857, df = 2, *p* < 0.001). Young farmers exhibited the highest proportion of high knowledge (98.1%, 51/52), compared with adult farmers (28.9%, 22/76) and older farmers (27.3%, 6/22). Educational status showed the strongest association with knowledge level (FFH exact test = 173.213, *p* < 0.001). All respondents with primary (100%, 66/66) or secondary education (100%, 10/10) demonstrated high knowledge, whereas only 4.1% (3/74) of illiterate respondents were classified as having high knowledge. In contrast, no statistically significant associations were observed between knowledge level and gender (*χ* ^2^ = 0.107, df = 1, *p* = 0.744), district of residence (*χ* ^2^ = 0.695, df = 2, *p* = 0.706), or distance to the nearest veterinary facility (FFH exact test = 1.278, *p* = 0.746). Knowledge levels were comparably distributed across these variables. The detailed distribution of knowledge scores across all sociodemographic and spatial variables is presented in Table 2 (see also Appendix 2 for observed and expected cell counts of all the crosstabs).

**TABLE 2:** Association of sociodemographic and spatial factors with knowledge of equine endoparasites among livestock farmers (*n* = 150).

Variable	Category	Total *n* (%)	Knowledge level		Chi‐square test			Fisher–Freeman–Halton exact test	
			Moderate *n* (%)	High *n* (%)	*χ* ^2^	df	*p* value	Statistic	*p* value
Gender	Male	120 (80.0)	56 (37.3)	64 (42.7)	0.107	1	0.744	—	—
	Female	30 (20.0)	15 (10.0)	15 (10.0)					

Age	Young	52 (34.7)	1 (0.7)	51 (34.0)	65.857	2	< 0.001^∗^	—	—
	Adult	76 (50.7)	54 (36.0)	22 (14.7)					
	Old	22 (14.7)	16 (10.7)	6 (4.0)					

Education	Illiterate	74 (49.3)	71 (47.3)	3 (2.0)	—	—	—	173.213	< 0.001^∗^
	Primary	66 (44.0)	0 (0.0)	66 (44.0)					
	Secondary	10 (6.7)	0 (0.0)	10 (6.7)					

District	Dera	50 (33.3)	22 (14.7)	28 (18.7)	0.695	2	0.706	—	—
	Fogera	50 (33.3)	26 (17.3)	24 (16.0)					
	Libokemkem	50 (33.3)	23 (15.3)	27 (18.0)					

Vet facility distance	< 1 km	8 (5.3)	4 (2.7)	4 (2.7)	—	—	—	1.278	0.746
	1–5 km	56 (37.3)	24 (16.0)	32 (21.3)					
	5–10 km	59 (39.3)	28 (18.7)	31 (20.7)					
	> 10 km	27 (18.0)	15 (10.0)	12 (8.0)					

Income source	Mixed farming	150 (100.0)	71 (47.3)	79 (52.7)	—	—	—	—	—

Overall	Total Farmers	150 (100.0)	71 (47.3)	79 (52.7)	—	—	—	—	—

^∗^indicates statistically significant association (*p* < 0.05).

### 3.3. Attitudes of Farmers Toward Equine Endoparasites

Respondents demonstrated a predominantly positive attitude toward the management of equine endoparasites. Overall, 66.7% (*n* = 100) were classified as having a good attitude, 4.0% (*n* = 6) as moderate, and 29.3% (*n* = 44) as poor (Table 3) (see also Appendix 2 for observed and expected cell counts of all the crosstabs).

**TABLE 3:** Association between demographic characteristics and attitude levels toward equine endoparasite control (*n* = 150).

Variable	Category	Total *n* (%)	Attitude level			Fisher–Freeman–Halton–Exact test	
			Good *n* (%)	Moderate *n* (%)	Poor *n* (%)	Statistic	*p* value
Gender	Male	120 (80.0)	79 (65.8)	4 (3.3)	37 (30.8)	1.443	0.490
	Female	30 (20.0)	21 (70.0)	2 (6.7)	7 (23.3)		

Age	Young	52 (34.7)	51 (98.1)	0 (0.0)	1 (1.9)	41.940	< 0.001^∗^
	Adult	76 (50.7)	38 (50.0)	5 (6.6)	33 (43.4)		
	Old	22 (14.7)	11 (50.0)	1 (4.5)	10 (45.5)		

Education	Illiterate	74 (49.3)	24 (32.4)	6 (8.1)	44 (59.5)	87.764	< 0.001^∗^
	Primary	66 (44.0)	66 (100.0)	0 (0.0)	0 (0.0)		
	Secondary	10 (6.7)	10 (100.0)	0 (0.0)	0 (0.0)		

District	Dera	50 (33.3)	31 (62.0)	4 (8.0)	15 (30.0)	5.632	0.207
	Fogera	50 (33.3)	31 (62.0)	2 (4.0)	17 (34.0)		
	Libokemkem	50 (33.3)	38 (76.0)	0 (0.0)	12 (24.0)		

Vet facility distance	< 1 km	8 (5.3)	5 (62.5)	1 (12.5)	2 (25.0)	4.845	0.532
	1–5 km	56 (37.3)	40 (71.4)	2 (3.6)	14 (25.0)		
	5–10 km	59 (39.3)	38 (64.4)	1 (1.7)	20 (33.9)		
	> 10 km	27 (18.0)	17 (63.0)	2 (7.4)	8 (29.6)		

Income source	Mixed farming	150 (100.0)	100 (66.7)	6 (4.0)	44 (29.3)	—	—

Overall	All farmers	150	100 (66.7)	6 (4.0)	44 (29.3)	—	—

^∗^indicates statistically significant association (*p* < 0.05).

Significant associations were observed between attitude and age as well as educational status. Age group was significantly associated with attitude (FFH exact test = 41.940, *p* < 0.001). Young farmers exhibited overwhelmingly positive attitudes, with 98.1% (51/52) classified as good, compared with 50.0% of adult (38/76) and 50.0% of older farmers (11/22). Educational status also demonstrated a strong association with attitude (FFH exact test = 87.764, *p* < 0.001). All farmers with primary (100%, 66/66) or secondary education (100%, 10/10) had good attitudes, whereas only 32.4% (24/74) of illiterate farmers exhibited a positive attitude, with the majority (59.5%, 44/74) classified as poor. No statistically significant associations were found between attitude level and gender (FFH exact test = 1.443, *p* = 0.490), district of residence (FFH exact test = 5.632, *p* = 0.207), or distance to the nearest veterinary facility (FFH exact test = 4.845, *p* = 0.532), indicating similar distribution of attitudes across these categories (Table 3).

### 3.4. Practices of Farmers Toward Equine Endoparasites

Farmers demonstrated a high level of practices toward the management of equine endoparasites. Overall, 91.3% (*n* = 137) were classified as having high practice levels, 6.0% (*n* = 9) as moderate, and 2.7% (*n* = 4) as low (Table 4) (see also Appendix 2 for observed and expected cell counts of all the crosstabs).

**TABLE 4:** Factors associated with practices in equine endoparasite management among livestock farmers (*n* = 150).

Variable	Category	Total *n* (%)	Practice level			Fisher–Freeman–Halton exact test	
			Low *n* (%)	Moderate *n* (%)	High *n* (%)	Statistic	*p* value
Gender	Male	120 (80.0)	2 (1.3)	5 (3.3)	113 (75.3)	6.031	0.050^∗^
	Female	30 (20.0)	2 (1.3)	4 (2.7)	24 (16.0)		

Age	Young	52 (34.7)	1 (0.7)	0 (0.0)	51 (34.0)	44.778	< 0.001^∗^
	Adult	76 (50.7)	0 (0.0)	0 (0.0)	76 (50.7)		
	Old	22 (14.7)	3 (2.0)	9 (6.0)	10 (6.7)		

Education	Illiterate	74 (49.3)	1 (0.7)	7 (4.7)	66 (44.0)	5.185	0.219
	Primary	66 (44.0)	2 (1.3)	2 (1.3)	62 (41.3)		
	Secondary	10 (6.7)	1 (0.7)	0 (0.0)	9 (6.0)		

Vet facility distance	< 1 km	8 (5.3)	0 (0.0)	0 (0.0)	8 (5.3)	12.243	0.021^∗^
	1–5 km	56 (37.3)	0 (0.0)	1 (0.7)	55 (36.7)		
	5–10 km	59 (39.3)	1 (0.7)	4 (2.7)	54 (36.0)		
	> 10 km	27 (18.0)	3 (2.0)	4 (2.7)	20 (13.3)		

District	Dera	50 (33.3)	1 (0.7)	2 (1.3)	47 (31.3)	1.492	0.836
	Fogera	50 (33.3)	2 (1.3)	4 (2.7)	44 (29.3)		
	Libokemkem	50 (33.3)	1 (0.7)	3 (2.0)	46 (30.7)		

Income source	Mixed farming	150 (100.0)	4 (2.7)	9 (6.0)	137 (91.3)	—	—

Overall	Total farmers	150 (100.0)	4 (2.7)	9 (6.0)	137 (91.3)	—	—

^∗^indicates statistically significant association (*p* < 0.05).

Significant associations were observed between practice levels and age, gender, and distance to the nearest veterinary facility. Age was strongly associated with practice (FFH exact test = 44.778, *p* < 0.001). Young and adult farmers exhibited high practice levels (98.1%, 51/52% and 100%, 76/76, respectively), while only 45.5% (10/22) of older farmers were classified as high. Distance to the nearest veterinary facility was also significantly associated with practice (FFH exact test = 12.243, *p* = 0.021). A clear gradient was observed: Farmers living within 5 km of a facility had 97%–100% high practice levels, whereas those living more than 10 km away had the lowest (74.1%, 20/27). Gender demonstrated a significant association with practice (FFH exact test = 6.031, *p* = 0.050). Male farmers had a higher proportion of high practice levels (94.2%, 113/120) compared to female farmers (80.0%, 24/30).

**FIGURE 3:**
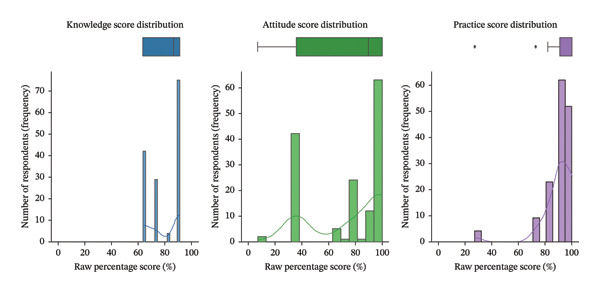
Distribution of raw percentage KAP scores among farmers (*n* = 150), presented using histograms and boxplots.

No statistically significant associations were found between practice levels and education (FFH exact test = 5.185, *p* = 0.219) or district of residence (FFH exact test = 1.492, *p* = 0.836), indicating similar distribution of practices across these variables. The complete distribution of practice levels across all demographic characteristics is presented in Table 4. Figure 3 further illustrates the distribution of raw percentage scores for KAP among respondents using histograms and boxplots.

### 3.5. Distribution of Responses to Knowledge Questions on Equine Endoparasites

All participants (100%, *n* = 150) correctly answered the first eight knowledge questions, indicating universal awareness of internal parasites in equines (Q1), their association with weight loss (Q2), diarrhea or soft dung (Q3), and weakness or reduced working ability (Q4). In addition, all respondents identified grazing grass or soil as the primary route of infection (Q5), recognized higher susceptibility in young animals (Q6), acknowledged that parasites may be present in apparently healthy equines (Q7), and reported awareness of fecal (dung) examination as a diagnostic method (Q8).

In contrast, lower levels of awareness were observed for items related to anthelmintic use and resistance. Only 72.0% (*n* = 108) of participants reported that administering the correct dose of deworming drugs is important (Q9). Awareness of anthelmintic resistance (AR) was reported by 50.0% (*n* = 75) of respondents (Q10). Similarly, 52.7% (*n* = 79) of participants recognized that repeated use of the same anthelmintic drug can reduce its effectiveness or efficacy (Q11). The distribution of responses for all 11 knowledge items is presented in Table 5.

**TABLE 5:** Farmers’ responses to knowledge assessment questions on equine internal parasites (*n* = 150).

No.	Knowledge assessment question	Response category	Frequency (*n*)	Percentage (%)
Q1	Have you ever heard about internal parasites (worms) in horses/donkeys/mules?	Yes	150	100.0

Q2	Do you think internal parasites can make equines lose weight?	Yes	150	100.0

Q3	Can internal parasites cause diarrhea or soft dung?	Yes	150	100.0

Q4	Can internal parasites cause weakness or poor working ability?	Yes	150	100.0

Q5	How do equines mostly get internal parasites?	Grazing grass/soil	150	100.0

Q6	Can young animals get more parasites than adults?	Yes	150	100.0

Q7	Can parasites be present even when the animal looks healthy?	Yes	150	100.0

Q8	Have you heard about testing dung (feces) to check for parasites?	Yes	150	100.0

Q9	Do you think giving the correct dose of deworming drug (medicine) is important?	No	42	28.0
		Yes	108	72.0

Q10	Have you heard that worms can become resistant (medicine no longer works well)?	No	75	50.0
		Yes	75	50.0

Q11	Do you think using the same deworming medicine many times can make it less effective?	No	71	47.3
		Yes	79	52.7

### 3.6. Frequency Distribution of Responses to Attitude Items Regarding Equine Endoparasites

The seven‐item attitude questionnaire assessed farmers’ perspectives on equine endoparasites, and the frequency and percentage of responses for each item are presented in Table 6. Nearly all respondents (98.7%) agreed that preventing equine endoparasites is necessary (A3). High agreement was also observed for the importance of being informed about equine endoparasites (A5: 70.7%) and for the benefits of controlling endoparasites for equine health (A6: 70.0%). A majority believed that endoparasites negatively affect equine productivity (A2: 66.0%) and constitute a serious health problem (A1: 68.0%).

**TABLE 6:** Frequency distribution of responses to attitude items regarding equine endoparasites (*n* = 150).

Item	Attitude statement	Disagree *n* (%)	Neutral *n* (%)	Agree *n* (%)
A1	Endoparasites (worms) are a serious health problem for my equids	44 (29.3)	4 (2.7)	102 (68.0)
A2	Endoparasites reduce the productivity (e.g., growth, condition, work output) of my equids	2 (1.3)	49 (32.7)	99 (66.0)
A3	Preventing equine endoparasites is necessary for me as an owner	0 (0.0)	2 (1.3)	148 (98.7)
A4	Preventing equine endoparasites is my responsibility as an owner	73 (48.7)	14 (9.3)	63 (42.0)
A5	It is important for me, as an owner, to stay informed about equine parasites and control methods	2 (1.3)	42 (28.0)	106 (70.7)
A6	Controlling equine endoparasites is beneficial for equine health	2 (1.3)	43 (28.7)	105 (70.0)
A7	Failure to control equine endoparasites can have serious consequences for my animals	44 (29.3)	31 (20.7)	75 (50.0)

Responses were more divided regarding personal responsibility for prevention. Almost half of the respondents (48.7%) disagreed that preventing equine endoparasites is their responsibility as owners (A4), while 42.0% agreed and 9.3% were neutral. Similarly, only half of respondents (50.0%) agreed that failure to control endoparasites can have serious consequences (A7), with 29.3% disagreeing and 20.7% neutral. Neutral responses were notably higher for A2 (32.7%), indicating some uncertainty about the impact of endoparasites on equine productivity. Items A5 and A6 also showed considerable neutral responses (28.0% and 28.7%, respectively).

Overall, across all seven items, 66.5% of responses reflected agreements, 17.6% were neutral, and 15.9% reflected disagreement. The seven attitude items demonstrated acceptable internal consistency (Cronbach’s *α* = 0.79). The composite attitude score, calculated as the mean of all seven items (possible range: 0–2), indicated moderate positive attitudes toward equine endoparasite control (*M* = 1.41, SD = 0.42).

### 3.7. Practices Adopted by Farmers for the Prevention and Control of Equine Endoparasites

The reported preventive practices are summarized in Table 7. Nearly all respondents (97.3%) reported using some method to prevent endoparasites (P2). The vast majority identified “good management” as their main preventive method (P3: 97.3%), which is consistent with the high reported frequencies of regular housing cleaning (P6: 97.3%) and manure removal (P7: 97.3%). Concurrently, most respondents dewormed their equines regularly (P4: 78.7%) and relied on a veterinary professional for this decision (P5: 79.3%). The predominant grazing systems were free grazing (P1: 49.3%) and semi‐intensive (P1: 46.7%).

**TABLE 7:** Equine endoparasite control practices among respondents (*n* = 150).

Item	Practice variable	Response category	Frequency (*n*)	Percentage (%)
P1	What grazing system do you use for your equines?	House keeping	6	4.0
		Free grazing	74	49.3
		Semi‐intensive	70	46.7

P2	Do you use any method to prevent equine endoparasites?	No	4	2.7
		Yes	146	97.3

P3	What is the main method used to prevent equine endoparasites?	Medication (deworming)	4	2.7
		Avoiding contact with other equines	0	0.0
		Good management (clean housing, manure removal, pasture hygiene)	146	97.3

P4	How often do you deworm your equine?	Never	0	0.0
		Occasionally (once a year or less)	32	21.3
		Regularly (≥ 2 times per year)	118	78.7

P5	Who decides or administers the deworming?	Self/traditional decision	31	20.7
		Veterinary professional	119	79.3

P6	Do you clean equine housing regularly?	No	4	2.7
		Yes	146	97.3

P7	Do you remove manure from housing or grazing areas?	No	4	2.7
		Yes	146	97.3

### 3.8. Univariable and Multivariable Binary Logistic Regression Analysis of Factors Influencing Farmers’ KAP on Equine Endoparasites

#### 3.8.1. Knowledge

The results of the logistic regression analyses examining factors associated with farmers’ knowledge of equine endoparasites are presented in Table 8. Overall, 79 farmers (52.7%) demonstrated good knowledge, whereas 71 (47.3%) exhibited poor knowledge. In the univariable analysis, the age group and education level were statistically significant (*p* < 0.001) predictors of knowledge. Among young farmers (*n* = 52), 98.1% (51/52) had good knowledge, compared to only 28.9% (22/76) of adult farmers and 27.3% (6/22) of old farmers. Consequently, both adult farmers (crude odds ratio [COR] = 0.01, 95% CI: 0.001–0.06, *p* < 0.001) and old farmers (COR = 0.01, 95% CI: 0.001–0.07, *p* < 0.001) had drastically lower odds of good knowledge compared to young farmers. A stark contrast was observed by education: 100% (76/76) of educated farmers had good knowledge versus 4.1% (3/74) of illiterate farmers. Accordingly, farmers with formal education had 57.6 times higher odds of good knowledge compared to illiterate farmers (COR = 57.6, 95% CI: 12.8–259.2, *p* < 0.001). Gender, district, and distance to the nearest veterinary facility showed no statistically significant association with knowledge level (*p* > 0.05 for all categories).

**TABLE 8:** Univariable and multivariable logistic regression analyses of factors associated with good knowledge of equine endoparasites among farmers (*n* = 150).

Variable	Category	Total *n* (%)	Knowledge level		Univariable analysis			Multivariable analysis (model: age + education)		
			Poor *n* (%)	Good *n* (%)	COR	95% CI	*p* value	AOR	95% CI	*p* value
Gender	Male	120 (80.0)	56 (46.7)	64 (53.3)	1.00	Reference	—	—	—	—
	Female	30 (20.0)	15 (50.0)	15 (50.0)	0.88	0.39–1.95	0.744	—	—	—

Age group	Young	52 (34.7)	1 (1.9)	51 (98.1)	1.00	Reference	—	1.00	Reference	—
	Adult	76 (50.7)	54 (71.1)	22 (28.9)	0.01	0.001–0.06	< 0.001^∗^	0.12	0.03–0.50	0.004^∗^
	Old	22 (14.7)	16 (72.7)	6 (27.3)	0.01	0.001–0.07	< 0.001^∗^	0.10	0.02–0.55	0.008^∗^

Education level	Illiterate	74 (49.3)	71 (95.9)	3 (4.1)	1.00	Reference	—	1.00	Reference	—
	Educated	76 (50.7)	0 (0.0)	76 (100.0)	57.6	12.8–259.2	< 0.001^∗^	15.8	3.4–73.8	< 0.001^∗^

District	Dera	50 (33.3)	22 (44.0)	28 (56.0)	1.00	Reference	—	—	—	—
	Fogera	50 (33.3)	26 (52.0)	24 (48.0)	0.73	0.33–1.59	0.424	—	—	—
	Libokemkem	50 (33.3)	23 (46.0)	27 (54.0)	0.92	0.42–2.03	0.841	—	—	—

Distance to vet facility	< 1 km	8 (5.3)	4 (50.0)	4 (50.0)	1.00	Reference	—	—	—	—
	1–5 km	56 (37.3)	24 (42.9)	32 (57.1)	1.33	0.30–5.88	0.704	—	—	—
	5–10 km	59 (39.3)	28 (47.5)	31 (52.5)	1.11	0.25–4.85	0.893	—	—	—
	> 10 km	27 (18.0)	15 (55.6)	12 (44.4)	0.80	0.17–3.89	0.782	—	—	—

*Note:* Model diagnostics for the multivariable logistic regression: Hosmer–Lemeshow goodness‐of‐fit *p* = 0.966; c‐statistic (AUC) = 0.996; Nagelkerke *R* ^2^ = 0.941; no multicollinearity detected (VIF = 1.012–1.648; tolerance = 0.607–0.988).

Abbreviations: AOR = adjusted odds ratio, CI = confidence interval, COR = crude odds ratio.

^∗^Statistically significant.

The multivariable logistic regression model for knowledge showed good calibration (Hosmer–Lemeshow *p* = 0.966) and excellent discrimination (c‐statistic, i.e., AUC = 0.996). The Nagelkerke *R* ^2^ was 0.941, indicating strong explanatory power. Furthermore, no evidence of multicollinearity was observed among the predictor variables, as all variance inflation factor (VIF) values were below 5 (range: 1.012–1.648), with tolerance values ranging from 0.607 to 0.988, all above the acceptable threshold of 0.1. Within this well‐fitting model, both age and education remained strong, independent predictors. The effect of age was attenuated but significant: Adult farmers had 88% lower odds (AOR = 0.12, 95% CI: 0.03–0.50, *p* = 0.004), and older farmers had 90% lower odds (AOR = 0.10, 95% CI: 0.02–0.55, *p* = 0.008) of good knowledge compared to young farmers. The association for education was also attenuated but remained highly significant; educated farmers had 15.8 times higher odds of good knowledge compared to illiterate farmers after adjusting for age (AOR = 15.8, 95% CI: 3.4–73.8, *p* < 0.001).

#### 3.8.2. Attitude

The results of the logistic regression analyses for factors associated with attitude toward equine endoparasites are presented in Table 9. Overall, 100 farmers (66.7%) showed a good attitude, while 50 (33.3%) demonstrated a poor attitude. In the univariable analysis, the age group and education level were statistically significant predictors of attitude. Among young farmers (*n* = 52), 98.1% (51/52) had a good attitude, compared to only 50.0% (38/76) of adult farmers and 50.0% (11/22) of old farmers. Compared to young farmers, both adult farmers (COR = 0.02, 95% CI: 0.003–0.15, *p* < 0.001) and old farmers (COR = 0.02, 95% CI: 0.002–0.17, *p* < 0.001) had drastically lower odds of a good attitude. A strong association was found with education: 100% (76/76) of educated farmers had a good attitude versus 32.4% (24/74) of illiterate farmers. Accordingly, farmers with formal education had 63.5 times higher odds of a good attitude compared to illiterate farmers (COR = 63.5, 95% CI: 15.0–269.0, *p* < 0.001). Gender, district, and distance to the nearest veterinary facility showed no statistically significant association with attitude level (*p* > 0.05 for all categories). Specifically, a good attitude was reported by 65.8% (79/120) of males and 70.0% (21/30) of females; by 62.0% (31/50) of farmers in both Dera and Fogera districts, and 76.0% (38/50) in Libokemkem; and by 62.5%–71.4% of farmers across different distance categories.

**TABLE 9:** Factors associated with attitude toward equine endoparasite control: univariable and multivariable logistic regression results (*n* = 150).

Variable	Category	Total *n* (%)	Attitude level		Univariable analysis			Multivariable analysis (model: age + district + education)		
			Poor *n* (%)	Good *n* (%)	COR	95% CI	*p* value	AOR	95% CI	*p* value
Gender	Male	120 (80.0)	41 (34.2)	79 (65.8)	1.00	Reference	—	—	—	—
	Female	30 (20.0)	9 (30.0)	21 (70.0)	1.21	0.51–2.88	0.665	—	—	—

Age group	Young	52 (34.7)	1 (1.9)	51 (98.1)	1.00	Reference	—	1.00	Reference	—
	Adult	76 (50.7)	38 (50.0)	38 (50.0)	0.02	0.003–0.15	< 0.001^∗^	0.015	0.002–0.120	< 0.001^∗^
	Old	22 (14.7)	11 (50.0)	11 (50.0)	0.02	0.002–0.17	< 0.001^∗^	0.013	0.001–0.119	< 0.001^∗^

Education level	Illiterate	74 (49.3)	50 (67.6)	24 (32.4)	1.00	Reference	—	—	—	—
	Educated	76 (50.7)	0 (0.0)	76 (100.0)	63.5	15.0–269.0	< 0.001^∗^	18.5	5.0–68.0	< 0.001^∗^

District	Dera	50 (33.3)	19 (38.0)	31 (62.0)	1.00	Reference	—	1.00	Reference	—
	Fogera	50 (33.3)	19 (38.0)	31 (62.0)	1.00	0.45–2.24	1.000	1.292	0.467–3.575	0.621
	Libokemkem	50 (33.3)	12 (24.0)	38 (76.0)	1.94	0.82–4.61	0.133	3.796	1.363–10.572	0.011^∗^

Distance to vet facility	< 1 km	8 (5.3)	3 (37.5)	5 (62.5)	1.00	Reference	—	—	—	—
	1–5 km	56 (37.3)	16 (28.6)	40 (71.4)	1.50	0.32–7.03	0.607	—	—	—
	5–10 km	59 (39.3)	21 (35.6)	38 (64.4)	1.09	0.24–5.00	0.916	—	—	—
	> 10 km	27 (18.0)	10 (37.0)	17 (63.0)	1.02	0.20–5.21	0.981	—	—	—

*Note:* Model diagnostics for the multivariable logistic regression: Hosmer–Lemeshow goodness‐of‐fit *p* = 1.000; c‐statistic (AUC) = 0.913; Nagelkerke *R* ^2^ = 0.680; no evidence of multicollinearity was observed (VIF = 1.067–1.636; tolerance = 0.611–0.937, all above the acceptable threshold of 0.1).

Abbreviations: AOR = adjusted odds ratio, CI = confidence interval, COR = crude odds ratio.

^∗^Statistically significant.

The multivariable logistic regression model demonstrated good fit and discrimination, with a Hosmer–Lemeshow goodness‐of‐fit of *p* = 1.000, c‐statistic (AUC) = 0.913, and Nagelkerke *R* ^2^ = 0.680. No evidence of multicollinearity was observed among predictors, as all VIF values were well below the conventional threshold of 5 (range: 1.067–1.636) and tolerance values ranged from 0.611 to 0.937, all above the acceptable threshold of 0.1, supporting the reliability of the model estimates.

After adjustment, the final multivariable model included age, education, and district. Age remained a strong predictor, although attenuated: Adult farmers had 98.5% lower odds (AOR = 0.015, 95% CI: 0.002–0.120, *p* < 0.001), and older farmers had 98.7% lower odds (AOR = 0.013, 95% CI: 0.001–0.119, *p* < 0.001) of a good attitude compared to young farmers. Educated farmers had 18.5 times higher odds of a good attitude compared to illiterate farmers after adjustment (AOR = 18.5, 95% CI: 5.0–68.0, *p* < 0.001). Farmers from Libokemkem had 3.8 times higher odds of a good attitude compared to those from Dera district (AOR = 3.796, 95% CI: 1.363–10.572, *p* = 0.011).

#### 3.8.3. Practice

The results of the logistic regression analyses for factors associated with good practice toward equine endoparasite control are presented in Table 10. Overall, 137 farmers (91.3%) reported good practices, while 13 (8.7%) reported poor practices. In the univariable analysis, gender, age group, and distance to the nearest veterinary facility were statistically significant predictors of practice. Good practice was reported by 94.2% (113/120) of male farmers compared to 80.0% (24/30) of female farmers. Female farmers had 75.2% lower odds of good practice compared to male farmers (COR = 0.248, 95% CI: 0.076–0.803, *p* = 0.020). There was a stark contrast by age: 98.1% (51/52) of young farmers, 100% (76/76) of adult farmers, and 45.5% (10/22) of old farmers reported good practices. Consequently, adult farmers had 81.8 times higher odds of good practice compared to young farmers (COR = 81.8, 95% CI: 5.5–368.0, *p* < 0.001), while old farmers had 98.4% lower odds (COR = 0.016, 95% CI: 0.002–0.140, *p* < 0.001). Greater distance was associated with lower odds of good practice: 100% (8/8) of farmers within 1 km, 98.2% (55/56) at 1–5 km, 91.5% (54/59) at 5–10 km, and 74.1% (20/27) of those living more than 10 km away reported good practices. Farmers living more than 10 km away had a significant 95% reduction in odds (COR = 0.05, 95% CI: 0.003–0.9, *p* = 0.042). Education level (89.2% vs. 93.4% good practice for illiterate and educated, respectively) and district (94.0%, 88.0%, and 92.0% good practice in Dera, Fogera, and Libokemkem, respectively) showed no significant association with practice level (*p* > 0.05 for all categories).

**TABLE 10:** Univariable and multivariable logistic regression analyses of factors associated with good practice toward equine endoparasites control (*n* = 150).

Variable	Category	Total *n* (%)	Practice level		Univariable analysis			Multivariable analysis (model: gender, age, distance)		
			Poor *n* (%)	Good *n* (%)	COR	95% CI	*p* value	AOR	95% CI	*p* value
Gender	Male	120 (80.0)	7 (5.8)	113 (94.2)	1.00	Reference	—	1.00	Reference	—
	Female	30 (20.0)	6 (20.0)	24 (80.0)	0.248	0.076–0.803	0.020^∗^	0.18	0.04–0.78	0.021^∗^

Age group	Young	52 (34.7)	1 (1.9)	51 (98.1)	1.00	Reference	—	1.00	Reference	—
	Adult ^a	76 (50.7)	0 (0.0)	76 (100.0)	81.8	5.5–368.0	< 0.001^∗^	54.5	4.8–620.0	< 0.001^∗^
	Old	22 (14.7)	12 (54.5)	10 (45.5)	0.016	0.002–0.140	< 0.001^∗^	0.02	0.002–0.22	0.001^∗^

Education level	Illiterate	74 (49.3)	8 (10.8)	66 (89.2)	1.00	Reference	—	—	—	—
	Educated	76 (50.7)	5 (6.6)	71 (93.4)	1.721	0.536–5.527	0.362	—	—	—

District	Dera	50 (33.3)	3 (6.0)	47 (94.0)	1.00	Reference	—	—	—	—
	Fogera	50 (33.3)	6 (12.0)	44 (88.0)	0.468	0.110–1.987	0.303	—	—	—
	Libokemkem	50 (33.3)	4 (8.0)	46 (92.0)	0.734	0.156–3.462	0.696	—	—	—

Distance to vet facility	< 1 km	8 (5.3)	0 (0.0)	8 (100.0)	1.00	Reference	—	1.00	Reference	—
	1–5 km	56 (37.3)	1 (1.8)	55 (98.2)	0.4	0.02–6.5	0.520	0.38	0.02–7.50	0.530
	5–10 km	59 (39.3)	5 (8.5)	54 (91.5)	0.2	0.01–3.8	0.280	0.18	0.01–3.60	0.260
	> 10 km	27 (18.0)	7 (25.9)	20 (74.1)	0.05	0.003–0.9	0.042^∗^	0.06	0.004–0.95	0.045^∗^

*Note:* Model diagnostics for the multivariable logistic regression: Hosmer–Lemeshow goodness‐of‐fit *p* = 0.802; c‐statistic (AUC) = 0.996; Nagelkerke *R* ^2^ = 0.758; no evidence of multicollinearity (VIF = 1.011–1.045; tolerance = 0.956–0.989, all above the acceptable threshold of 0.1).

Abbreviations: AOR = adjusted odds ratio, CI = confidence interval, COR = crude odds ratio.

^∗^Statistically significant.

The multivariable logistic regression model demonstrated good fit and excellent predictive performance, as indicated by a nonsignificant Hosmer–Lemeshow goodness‐of‐fit test (*p* = 0.802). The model showed outstanding discriminative ability with a c‐statistic (AUC) of 0.996 and explained a substantial proportion of the variance in the outcome (Nagelkerke *R* ^2^ = 0.758). In addition, no evidence of multicollinearity was detected among the independent variables, as all VIF values were below 5 (1.011–1.045) and tolerance values were high (0.956–0.989), all well above the acceptable threshold of 0.1. Overall, these diagnostics indicate an adequate model fit for inference.

**FIGURE 4:**
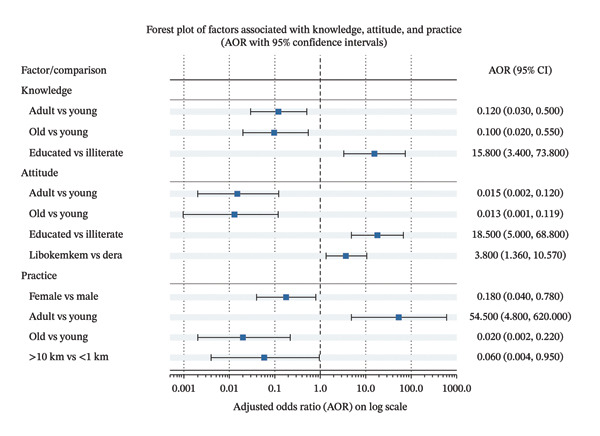
Forest plot showing statistically significant AORs with 95% CIs for factors associated with good KAP toward equine endoparasites control among farmers in South Gondar Zone, Northwest Ethiopia.

A multivariable model was constructed including age, gender, and distance to veterinary facility. After mutual adjustment, all three variables remained significant independent predictors. The effect of gender was attenuated: Female farmers had 82% lower odds of good practice compared to males (AOR = 0.18, 95% CI: 0.04–0.78, *p* = 0.021). The strong association for age persisted: Adult farmers had 54.5 times higher odds (AOR = 54.5, 95% CI: 4.8–620.0, *p* < 0.001), while old farmers had 98% lower odds (AOR = 0.02, 95% CI: 0.002–0.22, *p* = 0.001) of good practice compared to young farmers. For distance to facility, only the farthest category (> 10 km) remained significant, with 94% lower odds of good practice (AOR = 0.06, 95% CI: 0.004–0.95, *p* = 0.045). The attenuation of the age (adult) and gender (female) effects suggests partial confounding between these variables and distance to veterinary facility. Figure 4 presents a visual summary of the statistically significant AORs and corresponding 95% CIs identified in the final multivariable logistic regression models for KAP outcomes.

## 4. Discussion

### 4.1. Overall KAP of Farmers Regarding Equine Endoparasite

The current study revealed a notable pattern in which the proportion of farmers reporting high practice levels exceeded those with high knowledge or positive attitudes. This practice–knowledge gap contrast with the more typical pattern where knowledge exceeds practice, as seen in other Ethiopian livestock health studies [28, 29]. Interestingly, a recent KAP study from the East Gojjam Zone in the same region reported the inverse pattern: higher knowledge (57%) and attitude (69%) scores than practice (49%) regarding general livestock diseases [3]. This divergence suggests that the relationship between KAP domains is highly context‐specific and may depend on the particular health intervention, the study population, or measurement methods. In our case, the high practice level may indicate that farmers are implementing control measures based on traditional knowledge systems, veterinary prescriptions, or community norms without fully understanding the underlying principles, a dynamic also observed in equine parasite management in high‐income countries [30, 31].

#### 4.1.1. Education as the Primary Determinant

Education emerged as the strongest predictor of both knowledge (FFH exact test = 87.764, *p* < 0.001) and attitude (*p* < 0.001) in our study. The stark contrast between illiterate farmers (32.4% positive attitudes, 4.0% high knowledge) and literate farmers (100% positive attitudes and knowledge) aligns with and is powerfully reinforced by recent findings. Yizengaw et al. [3] demonstrated that illiteracy was the most profound barrier to good practice, with illiterate farmers being 96% less likely (AOR = 0.04) to engage in good livestock disease management practices compared to those with college education. This finding powerfully echoes Alemayehu et al. [28], who reported significantly lower knowledge of internal parasites among illiterate farmers (OR = 0.08, *p* < 0.001). Together, these studies underscore that basic literacy is a fundamental enabler not only for accessing and understanding veterinary information [32] but, also more critically, for translating that understanding into sustained, effective action.

#### 4.1.2. Age‐Related Patterns and Generational Differences

Young farmers in our study demonstrated significantly better outcomes across all KAP domains, a finding supported by research on vaccine adoption in Ethiopia where younger farmers showed greater knowledge and acceptance [33]. The new study adds a nuanced layer to this generational analysis. While Yizengaw et al. [3] found that younger age (< 30 years) was associated with a less desirable attitude compared to adults (30–45 years); they affirm that knowledge and practice are shaped by experience and exposure. This suggests that attitudes may follow a different life‐course trajectory than knowledge or practice, potentially influenced by risk perception, economic pressures, or the division of labor within households. Similar patterns have been documented, where age significantly influenced disease recognition and adoption of veterinary intervention behavior [33, 34], indicating that age remains a key sociodemographic factor, albeit with complex effects across different KAP dimensions.

#### 4.1.3. Gender and Implementation Challenges

The borderline significant gender difference in practice levels (FFH exact test = 6.031, *p* = 0.050) suggests potential gender‐based disparities in resource access. This aligns with documented evidence from Ethiopia showing that women livestock owners often face greater constraints in accessing veterinary services, credit, and decision‐making authority [35, 36]. On the other hand, gender was not a significant predictor of knowledge or attitude. This aligns with the findings of Yizengaw et al. [3], although the generalizability of their result might be limited by a predominantly male sample (88%). This skewed sample composition itself reflects broader, systemic gender disparities in livestock ownership and decision‐making authority within the region. Therefore, the persistent, though borderline, significance in our study emphasizes the continued importance of employing gender‐sensitive approaches in extension program design to ensure equitable access.

#### 4.1.4. Veterinary Access and Spatial Inequality

Distance to veterinary facilities showed a significant association with practice levels (FFH exact test = 12.243, *p* = 0.021), with farmers living > 10 km away having substantially lower practice levels. This spatial gradient reflects broader veterinary service access challenges in rural Ethiopia [35, 37]. The conceptual framework helps explain how geographic, economic, and social factors interact to limit access [38, 39]. The study by Yizengaw et al. [3] expands this understanding from geographic to qualitative access, revealing that 55% of farmers were dissatisfied with the availability and coverage of veterinary services in their area. This indicates that the “distance” barrier is not merely spatial but also encompasses perceived service quality, reliability, and affordability. Effective intervention must therefore address both the physical proximity of services and the capacity and responsiveness of the veterinary system.

### 4.2. Distribution of Responses to Knowledge Questions on Equine Endoparasites

This knowledge assessment reveals a striking dichotomy in the understanding of equine parasitology, with near‐universal awareness of basic clinical concepts coexisting with substantial deficiencies in comprehension of AR. This pattern of asymmetric knowledge mirrors broader challenges observed in veterinary and livestock health education across different geographical and production contexts.

The exceptional performance on fundamental questions (100% correct for eight of eleven items) represents an unusually high knowledge level when compared to similar structured assessments in other settings. While studies among equine owners in Europe and North America report correct response rates of 65%–85% for questions on clinical signs and transmission [8, 40], this near‐perfect score is particularly notable. Interestingly, this level of comprehensive basic knowledge exceeds that found in broader livestock health assessments. For example, a large cross‐sectional KAP study among mixed livestock owners in the East Gojjam Zone of Ethiopia—a setting with different husbandry systems but similar reliance on animal health for livelihoods—found that 72.6% of participants had adequate general knowledge of livestock diseases, with specific gaps in understanding transmission pathways and prevention details [3]. The unanimous recognition of subclinical infection potential in our study is a crucial finding, as this concept forms the foundation for moving beyond symptomatic treatment to evidence‐based control. The Ethiopian study similarly identified that while awareness of disease was high, knowledge of specific preventive measures was more variable, suggesting that practical, observable concepts are more readily absorbed than preventive principles across different farming communities [3].

The concerning knowledge gaps regarding AR, with only 50% of participants demonstrating awareness, align with patterns observed in other livestock sectors and regions. Studies among horse owners in the United Kingdom (55%–65% awareness) ([41]) and Australia (45%–60%) ([42]) report comparable figures, indicating that this is a widespread issue rather than a localized problem. The marginal improvement in understanding that repeated anthelmintic use reduces efficacy (52.7%) compared to general AR awareness suggests that participants may recognize practical consequences without grasping the underlying biological mechanisms—a pattern also seen in broader livestock disease management where farmers often understand treatment failure symptoms but not their etiology [3]. This superficial understanding of complex health concepts appears common across livestock systems; the Ethiopian study similarly found that while 64% of farmers recognized vaccination as important, only 45% knew about appropriate vaccination intervals, mirroring our finding of partial comprehension without technical depth [3].

The relative strength in recognizing dosage importance (72% correct) compared to AR concepts likely reflects traditional veterinary extension messages emphasizing correct administration for efficacy and safety over sustainable use principles. This educational focus on immediate outcomes rather than long‐term consequences may explain the current knowledge disparity. The finding that nearly one‐third of respondents did not recognize the importance of precise dosing suggests that additional educational needs beyond resistance awareness alone. This parallels findings from the Ethiopian study where significant proportions of farmers engaged in risky practices (like improper carcass disposal) despite generally good disease awareness, highlighting a persistent knowledge–behavior gap [3].

The observed knowledge distribution pattern, where respondents scored high on basic questions but showed more variation on advanced concepts, reflects findings from other veterinary parasitology studies, such as those on small ruminants [43] and companion animals [44]. Consistent with Ethiopian KAP research, factors like education level and intensity of the farming system emerged as significant predictors of knowledge; these same factors are likely to influence equine health management as well [3]. This knowledge gap is especially concerning given the high risk of AR in equine parasites. Globally, benzimidazole resistance is well documented in cyathostomins, and macrocyclic lactone resistance is increasingly reported in *Parascaris* spp. [45, 46], making the observed deficiencies in owner knowledge particularly significant.

Several methodological considerations should inform interpretation. The uniform 100% correct response rate for basic questions could indicate genuine high knowledge but may also reflect ceiling effects limiting discrimination or potential social desirability bias. The cross‐sectional design precludes the assessment of knowledge evolution. Furthermore, as noted in the Ethiopian study, questionnaire responses may not fully capture nuanced understanding or practical capabilities [3].

The educational implications are considerable. The solid foundational knowledge among owners provides a rare and advantageous foundation for teaching more complex topics in veterinary education. Nevertheless, the specific knowledge gaps observed suggest that conventional approaches have failed to adequately communicate how AR develops and how drugs should be used sustainably. Future knowledge dissemination should emphasize: (i) a clear explanation of the biological mechanisms behind AR, (ii) the distinction between a treatment’s immediate efficacy and its long‐term sustainable use, and (iii) the integration of resistance principles into practical day‐to‐day management. These efforts must also take into account the socioeducational determinants of knowledge acquisition identified in broader livestock research, where formal education and farming system intensity significantly influence knowledge levels [3].

### 4.3. Attitude Items Assessing Farmers’ Perceptions of Equine Endoparasites

The findings of this study revealed a generally positive attitude among equine owners toward the importance of managing equine endoparasites. This aligns with broader trends observed in livestock disease management within Ethiopia and similar contexts. A strong consensus (98.7%) on the necessity of prevention mirrors the high levels of concern and perceived severity of animal diseases reported in other KAP surveys. For instance, Yizengaw et al. [3] found that 83% of livestock owners in the East Gojjam Zone acknowledged the significant economic and zoonotic importance of diseases, while Bahiru et al. [47] reported that a majority of respondents in the Amhara Region held a serious concern about rabies. This suggests that across different disease domains, Ethiopian livestock owners recognize health threats as significant [3, 47].

The high percentage of respondents who agreed on the importance of being informed (A5: 70.7%) and the benefits of control for animal health (A6: 70.0%) further reflects an openness to knowledge and modern veterinary practices. This favorable disposition is a critical foundation for educational interventions and is consistent with findings from studies on other preventive measures, such as vaccination, where farmers often express positive attitudes despite gaps in practice [48, 49].

#### 4.3.1. The Critical Attitude–Practice Gap and Contradictory Beliefs

Despite the overall positive orientation, a deeper analysis revealed significant and contradictory gaps between generalized beliefs and specific, actionable attitudes. The most striking contradiction lies between the near‐universal agreement that “preventing equine endoparasites is necessary” (A3: 98.7%) and the markedly lower agreement that “failure to control can have serious consequences” (A7: 50.0% agree). This indicates that while prevention is viewed as a normative good, a substantial portion of owners may underestimate the actual risk and impact of parasitism, perceiving it as a routine management issue rather than a serious health threat. This attenuated risk perception is a likely barrier to the adoption of more vigilant control strategies.

Furthermore, the study uncovered a critical gap in the attribution of responsibility. Almost half of the respondents (48.7%) disagreed that preventing endoparasites was *their personal responsibility* as an owner (A4). This dissonance, recognizing a problem but externalizing its solution, has been documented in other One Health contexts. For example, studies on antimicrobial use revealed that farmers might acknowledge resistance as a problem while attributing responsibility to veterinarians or the pharmaceutical industry [50]. Similarly, in rabies prevention, communities might identify the disease threat but not see the vaccination of their own dogs as a personal duty [47]. This external locus of control directly undermines the translation of positive general attitudes into sustained, owner‐driven preventive practices.

#### 4.3.2. Explanatory Factors and Barriers Underlying Attitudinal Patterns

The discrepancy between high general attitude scores and lower scores on specific, responsibility‐laden items can be attributed to a confluence of structural, educational, and sociocultural barriers identified in the broader literature.

Access to Services and Economic Constraints: Positive attitudes often fail to materialize into action when access to veterinary guidance, affordable anthelmintics, or diagnostic tools is limited. Yizengaw et al. [3] reported that 55% of livestock owners were dissatisfied with available veterinary services, a key barrier to implementing knowledge. The cost of drugs and treatments is frequently cited as a limiting factor, making regular, evidence‐based deworming economically challenging for many owners [51, 52].

Knowledge Gaps and Misconceptions: A positive attitude toward “control” is ineffective if underpinned by incorrect knowledge. The reliance on traditional treatments or calendar‐based deworming in the face of widespread AR exemplifies this. Studies show that while farmers may have a positive attitude toward deworming, their knowledge of parasite life cycles, resistance mechanisms, and the value of fecal egg counts (FECs) is often inadequate [5]. This technical knowledge gap prevents the effective operationalization of their positive intent.

Sociocultural Norms and Trust: Community practices and trust in information sources profoundly shape attitudes. The use of traditional remedies alongside modern medicine, as noted by Yizengaw et al. [3], indicates a pragmatic or culturally embedded attitude toward treatment that may not always align with scientific recommendations. Attitudes are also shaped by trust in extension agents and peers; ineffective past experiences or conflicting advice can erode confidence in recommended practices [53].

#### 4.3.3. Influence of Demographic Factors on Attitudes

Consistent with other KAP studies, this research suggests that attitudes are not uniform across demographics. The analysis by Yizengaw et al. [3] and others has consistently shown that higher educational attainment is a strong predictor of more informed and proactive health attitudes [54, 55]. In the context of equine endoparasites, educated owners might be more likely to perceive themselves as responsible agents (A4) and understand the serious consequences of inaction (A7), due to better access to and comprehension of technical information. Furthermore, the role of gender and farming system (e.g., intensive vs. extensive) noted in the broader livestock literature should be investigated, as these factors influence exposure to information, resource control, and risk perception, all of which shape disease management attitudes [52].

### 4.4. Practices Adopted by Farmers for the Prevention and Control of Equine Endoparasites

This study revealed a profile of equine endoparasite control practices characterized by near‐universal engagement in prevention, a strong reported reliance on biosecure “good management” and frequent, professionally guided anthelmintic use. Placing these findings within the global and regional context of equine parasitology emphasizes both encouraging trends and critical areas for intervention.

#### 4.4.1. High Reported Preventive Engagement: Contrast With Regional and Global Benchmarks

The overwhelming majority of respondents (97.3%) reported using some method to prevent endoparasites (P2), and similarly high proportions reported regular cleaning (P6) and manure removal (P7). This level of claimed engagement is notably higher than that found in broader livestock KAP studies within Ethiopia. For instance, Yizengaw et al. [3] reported that only 49% of livestock owners in the East Gojjam Zone demonstrated good disease‐prevention practices, with poor veterinary access and resource constraints cited as key barriers. This contrast suggests that the sampled equine owners might represent a more resourceful or motivated subset, or those equines, as high‐value working animals, receive prioritized care. Furthermore, the emphasis on manure management contrasts with older studies in pastoral systems, where such practices were rarely adopted due to labor constraints and different management paradigms [56]. However, this high self‐reported adherence must be interpreted cautiously. Social desirability biases, where respondents provide answers they believe are expected, are a well‐documented limitation in KAP surveys [9]. The consistent ∼97% “yes” rate across multiple practice items (P2, P6, P7) might partially reflect this bias, indicating a need for future studies to complement self‐reporting with observational validation.

#### 4.4.2. The “Good Management” Paradigm and Its Coexistence With Routine Deworming

A central and insightful finding is the identification of “good management (clean housing, manure removal, pasture hygiene)” as the main prevention method by 97.3% of respondents (P3), while 78.7% simultaneously deworm their equines regularly (P4). This presents an apparent paradox but reflects a nuanced understanding of integrated control. It suggests that owners conceptually prioritize biosecurity—aiming to reduce environmental contamination and break the parasite life cycle—as their foundational strategy, while using anthelmintics as a routine supporting measure. This aligns with modern parasitology principles, which advocate for sustainable control anchored in management, with strategic anthelmintic use as a component [57]. However, the critical divergence lies in the definition of “strategic.” The high frequency of deworming (≥ 2 times per year) reported here is characteristic of traditional, calendar‐based programs, which are globally recognized as a primary driver of AR [8]. Studies from the United Kingdom and Ireland have similarly found that despite awareness of resistance, many horse owners continue with frequent, interval‐based deworming without diagnostic guidance [58]. Therefore, while the reported philosophy is advanced, the execution of the chemical component may still adhere to outdated practices, emphasizing a vital gap between knowledge of principles and application of evidence‐based techniques.

#### 4.4.3. Veterinary Guidance as a Cornerstone and Opportunity

A highly positive finding is that 79.3% of respondents rely on a veterinary professional to decide or administer deworming (P5). This rate of professional engagement is comparable to or higher than figures from European contexts; for example, a large‐scale U.K. study found that 60.7% of horse owners sought worming advice from their veterinarian, and those who did were significantly more likely to adopt targeted treatment protocols based on diagnostic testing [59]. This finding is supported by a 2025 national survey in France, which concluded that veterinarians were the main source of information and advice for implementing deworming protocols [60]. This strong veterinary–client relationship is a significant asset and represents the most potent conduit for improving practice. It indicates a high level of trust and access, which can be leveraged to transition owners from routine calendar deworming to targeted treatment protocols based on FEC monitoring.

The current reliance on veterinarians for administration provides a direct opportunity for extension. Veterinary professionals are uniquely positioned to educate clients on FEC‐based strategies, the importance of correct dosing, and the perils of under‐dosing and resistance. As noted by Seyoum et al. [61] in their study of cart horses in Ethiopia, AR is an emerging threat, making the shift from purely frequency‐based to evidence‐based deworming not just ideal but imperative for sustainable control.

### 4.5. Univariable and Multivariable Binary Logistic Regression Analysis of Factors Influencing Farmers’ KAP on Equine Endoparasites

#### 4.5.1. Determinants of Knowledge and Attitude: The Primacy of Education and Youth

The final multivariable model for knowledge, adjusting for age and education, explained a substantial proportion of variance. After adjustment, the effect sizes for age, while remaining highly significant, were attenuated: Adult and old farmers had 88% (AOR = 0.12) and 90% (AOR = 0.10) lower odds of good knowledge than young farmers, respectively. The independent effect of formal education remained powerful, with educated farmers having 15.8 times higher odds (AOR = 15.8) of good knowledge. This indicates education as the cornerstone of knowledge acquisition, aligning with multivariable findings from similar contexts. For instance, Yizengaw et al. [3] in East Gojjam, Ethiopia, found that illiterate livestock owners had 76% lower odds (AOR = 0.24) of adequate disease knowledge than those with college education. This pattern is globally consistent; recent evidence from Pakistan indicated that while education boosts basic awareness, it leads to 18.8 times higher odds (AOR = 18.8) of implementing valid agricultural practices [62]. Similarly, studies in Nigeria confirmed that higher education is the primary predictor of risk perception, with those lacking tertiary qualifications having up to 14.37 times higher odds (COR = 14.37) of inadequate disease knowledge [63]. These findings reinforce the universal role of literacy in enhancing both agricultural awareness and the technical application of disease‐prevention measures.

A nearly identical pattern was observed for attitudes. In the multivariable model, the strong protective effect of youth and education persisted, with educated farmers having 18.5 times higher odds (AOR = 18.5) of a desirable attitude. The significant independent effect of residing in Libokemkem district (AOR = 3.8) highlights the crucial influence of local, contextual factors such as the intensity of past extension campaigns, which can shape community‐wide attitudes beyond individual characteristics.

Younger age was consistently associated with better knowledge and attitudes. While this aligns with studies showing that younger farmers are more receptive to new information and formal training (Refs. [64–66]), it contrasts with research on clinically apparent livestock diseases where experiential knowledge among older farmers confers an advantage [67]. This divergence suggests that endoparasite control, characterized by subclinical infections and complex life cycles, relies more heavily on formal education than on experience alone.

#### 4.5.2. Determinants of Practice: A Distinct Model Revealing the Knowledge–Practice Gap

The multivariable model for practice revealed a fundamentally different set of independent predictors, starkly illustrating the knowledge–practice gap. Crucially, education was not a significant predictor in the final model. This demonstrates that knowing what to do is insufficient for implementation, a well‐documented phenomenon in global health [68]. This finding adds critical nuance to the work of Yizengaw et al. [3], who, in contrast, found education to be the strongest predictor of practice for general livestock diseases (AOR = 0.04 for illiterate individuals). This divergence suggests that the barriers to acting on knowledge about equine‐specific care might be even more heavily governed by noneducational constraints than those for general livestock management.

Instead, practice was governed by factors of access and socioeconomic agency. The strong effect of gender persisted, with female farmers having 82% lower odds (AOR = 0.18) of good practice than males, likely reflecting systemic inequalities in resource control, especially in access to training, veterinary services, and animal health resources—patterns observed in other livestock health and zoonoses KAP studies where male farmers had higher odds of satisfactory knowledge and practices [64, 66, 69]. The nonlinear effect of age was pronounced: Adult farmers had 54.5 times higher odds (AOR = 54.5) of good practice than young farmers, while old farmers had 98% lower odds (AOR = 0.02). Finally, access to veterinary services was a decisive independent barrier. Farmers living more than 10 km from a facility had 94% lower odds (AOR = 0.06) of good practice, confirming geographic access as a non‐negotiable prerequisite for action [62].

### 4.6. Strengths of the Study

This study has several strengths. First, to the best of our knowledge, this is the first equine‐specific KAP study on endoparasite control conducted (in the South Gondar Zone, Northwest Ethiopia), thereby addressing a critical evidence gap for targeted interventions. Second, we employed a representative multistage probability sampling approach covering three districts with a sample size of 150 respondents, enhancing the generalizability of our findings to the broader equine‐owning population in the region. Third, data collection was carried out by six trained diploma‐level veterinary technicians, which minimized interviewer bias and ensured consistent administration of the questionnaire. Fourth, we performed robust multivariable logistic regression adjusting for key potential confounders (including age, education, distance to veterinary facility, and district), allowing us to identify independent predictors of good knowledge, positive attitude, and good practice.

### 4.7. Limitations of the Study

This study has several limitations that should be considered when interpreting the findings. First, the cross‐sectional design captures data at a single time point, which precludes establishing causal relationships; the observed associations are correlational rather than causal. Second, reliance on self‐reported data through interviewer‐administered questionnaires may introduce social desirability bias, particularly for practice‐related questions. This could partially explain the unusually high proportion (91.3%) of respondents classified as having “good practice”—a figure that should be interpreted with caution. Third, some multivariable logistic regression models produced sparse data, as evidenced by extremely wide CIs (e.g., AOR = 54.5, 95% CI: 4.8–620). These imprecise estimates likely reflect small cell counts and possible model overfitting, suggesting that several findings should be considered exploratory rather than definitive. Fourth, the sampling was limited to three districts in the South Gondar Zone, which may restrict generalizability to other regions with different agroecological conditions or farming systems. Fifth, the absence of objective parasitological measurements (e.g., FECs) means that reported practices could not be validated against actual parasite burdens or treatment outcomes. Despite these limitations, the study provides valuable baseline data for designing targeted interventions in the area, and we have fully addressed reproducibility by providing the complete questionnaire as Appendix 1.

## 5. Conclusions and Recommendations

This study highlights that age and education are key determinants of farmers’ KAP toward equine endoparasite control in South Gondar Zone, Ethiopia. Young and educated farmers were more likely to possess good knowledge and demonstrate positive attitudes, emphasizing the critical role of formal education in shaping awareness and perceptions of parasite management. Similarly, adult farmers exhibited better practical management practices, whereas females and farmers residing farther from veterinary services were less likely to implement appropriate practices, underscoring the influence of gender and accessibility on behavioral outcomes. Notably, the study revealed a relatively high level of good practices (91.3%) despite only moderate levels of knowledge, a finding that challenges the conventional KAP assumption that improved knowledge necessarily translates into better practice. These novel findings provide important evidence for the development of regionally appropriate and targeted extension programs tailored to the local context of equine parasite control. These findings suggest that targeted educational programs, with an emphasis on older and less‐educated farmers, alongside improved accessibility to veterinary services, could enhance parasite control practices, reduce infection risks, and improve equine health and productivity. Future interventions should integrate community‐based training, gender‐sensitive approaches, and strategic placement of veterinary resources to ensure equitable access and sustained behavioral change. Collectively, the results provide actionable insights for policymakers, extension agents, and animal health professionals to optimize equine parasite management in similar rural settings.

## Author Contributions

Habtamu Alemayehu: conceptualization, methodology, investigation, data curation, formal analysis, and writing–original draft. Tewodros Alemneh: conceptualization, methodology, supervision, validation, formal analysis, resources, software, visualization, writing–original draft, and writing–review and editing. Zewdu Seyoum: methodology, investigation, data curation, validation, and writing–review and editing. Moges Maru: supervision, validation, and writing–review and editing. Abrham Ayele: conceptualization, supervision, validation, and writing–review and editing. Mastewal Birhan: supervision, validation, and writing–review and editing.

## Funding

This work did not receive any grant from funding agencies in the public, commercial, or not‐for‐profit sectors.

## Ethics Statement

This study was conducted in accordance with established ethical standards for research involving human participants. Ethical clearance was obtained from the Institutional Review Board of the University of Gondar, College of Veterinary Medicine and Animal Sciences (Ref. No. CVMASc/UoG: 107/2022). Official authorization was further secured from the South Gondar Zone Administration and the respective District Livestock and Fishery Resources Development Offices prior to data collection. All participants were fully informed about the objectives, procedures, potential benefits, and possible risks of the study. Written informed consent was obtained from each participant before enrollment. Participants were assured of the confidentiality of the information provided, and their right to decline participation or withdraw from the study at any stage without any consequences was strictly respected.

## Conflicts of Interest

The authors declare no conflicts of interest.

## Supporting Information

Additional supporting information can be found online in the Supporting Information section.

## Supporting information


**Supporting Information 1** Appendix 1: complete questionnaire.

**Supporting Information 2** Appendix 2: full crosstabs with observed and expected cell counts for all χ²/ FFH exact tests.

**Supporting Information 3** STROBE‐R3

## Data Availability

The data used to support the findings of this study are publicly available in the Mendeley Data repository at https://doi.org/10.17632/2pgfhjw7ch.1.
