# Supplementary material for: Assessment of Knowledge, Attitudes, and Preventive Practices for Equine Endoparasite Control Among Livestock Owners in South Gondar Zone, Northwest Ethiopia
Source: Vet Med Int. 2026 Jul 22;2026:5535645. doi: 10.1155/vmi/5535645 (PMC13389813; doi:10.1155/vmi/5535645)
Supplement: Supplementary file 1 — Supporting Information 1 Appendix 1: complete questionnaire. [file VMI-2026-5535645-s001.docx]

**Appendix 1: Complete Questionnaire**

**Study Title:** *Assessment of Knowledge, Attitudes, and Preventive Practices for Equine Endoparasites Control among Livestock Owners in South Gondar Zone, Northwest Ethiopia: A Community-Based Cross-Sectional Study*

**Data collection period:** From September 2023 to July 2024
**Language:** Amharic (translated to English for analysis)
**Mode of administration:** Face‑to‑face interview by trained data collectors

**INSTRUCTIONS FOR THE INTERVIEWER**

Read each question exactly as written. Do not lead the respondent. Circle or tick the appropriate answer. All responses are confidential.

**PART A: SOCIO‑DEMOGRAPHIC CHARACTERISTICS OF RESPONDENTS**

| **Variable** | **Question Wording** | **Response Options** |
| --- | --- | --- |
| Name | Name of participant | _______________________________________________ (optional) |
| Gender | Gender | ☐ Male ☐ Female |
| Age | Age | ________ years (coded later as Young/Adult/Old) |
| Village | Name of village | ________________________________________________ |
| Kebele | Kebele | __________________________________ |
| District | District | ☐ Dera ☐ Fogera ☐ Libokemkem |
| Date | Date of interview | _________________________ |
| Education Level | What is your level of education? | ☐ Illiterate ☐ Primary ☐ Secondary ☐ Higher |
| Main Income Source | What is your main source of income? | ☐ Mixed farming ☐ Crop production ☐ Livestock rearing ☐ Other: ________________________________________ |
| Distance to Vet Facility | What is your estimated distance to the nearest veterinary facility? | ☐ < 1 km ☐ 1-5 km ☐ 5-10 km ☐ > 10 km |

**PART B: KNOWLEDGE OF EQUINE ENDOPARASITES (K1-K11)**

**INSTRUCTIONS: Answer each Question based on Your Knowledge. The Interviewer will Record Your Response.**

| **Item** | **Question Wording** | **Response Options** | **Good Knowledge (Score = 1)** |
| --- | --- | --- | --- |
| K1 | Have you ever heard about internal parasites (worms) in horses/donkeys/mules? | ☐ No ☐ Yes | Yes |
| K2 | Do you think internal parasites can make equines lose weight? | ☐ No ☐ Yes ☐ Don’t know | Yes |
| K3 | Can internal parasites cause diarrhea or soft dung? | ☐ No ☐ Yes ☐ Don’t know | Yes |
| K4 | Can internal parasites cause weakness or poor working ability? | ☐ No ☐ Yes ☐ Don’t know | Yes |
| K5 | How do equines mostly get internal parasites? | ☐ Grazing grass/soil ☐ Dirty water ☐ Other animals ☐ Don’t know | Grazing grass/soil; Dirty water |
| K6 | Can young animals get more parasites than adults? | ☐ No ☐ Yes ☐ Don’t know | Yes |
| K7 | Can parasites be present even when the animal looks healthy? | ☐ No ☐ Yes ☐ Don’t know | Yes |
| K8 | Have you heard about testing dung (feces) to check for parasites? | ☐ No ☐ Yes | Yes |
| K9 | Do you think giving the correct dose of deworming medicine is important? | ☐ No ☐ Yes ☐ Don’t know | Yes |
| K10 | Have you heard that worms can become resistant (medicine no longer works well)? | ☐ No ☐ Yes | Yes |
| K11 | Do you think using the same deworming medicine many times can make it less effective? | ☐ No ☐ Yes ☐ Don’t know | Yes |

Knowledge scoring: each correct answer = 1 point. Total score = sum of K1-K11 (range 0-11). Categorization based on percentage score: Poor knowledge = 0-5 correct (0-50%); Moderate knowledge = 6-8 correct (51-74%); Good knowledge = 9-11 correct (75-100%).

**PART C: ATTITUDES TOWARDS EQUINE ENDOPARASITES (A1-A7)**

**INSTRUCTIONS:** **Indicate Your Level of Agreement with each Statement.**

| **Item** | **Statement** | **Response Options** | **Scoring** |
| --- | --- | --- | --- |
| A1 | Endoparasites (worms) are a serious health problem for my equids | ☐ Disagree ☐ Neutral ☐ Agree | Disagree = 0, Neutral = 1, Agree = 2 |
| A2 | Endoparasites reduce the productivity (e.g., growth, condition, work output) of my equids | ☐ Disagree ☐ Neutral ☐ Agree | Disagree = 0, Neutral = 1, Agree = 2 |
| A3 | Preventing equine endoparasites is necessary for me as an owner | ☐ Disagree ☐ Neutral ☐ Agree | Disagree = 0, Neutral = 1, Agree = 2 |
| A4 | Preventing equine endoparasites is my responsibility as an owner | ☐ Disagree ☐ Neutral ☐ Agree | Disagree = 0, Neutral = 1, Agree = 2 |
| A5 | It is important for me, as an owner, to stay informed about equine parasites and control methods | ☐ Disagree ☐ Neutral ☐ Agree | Disagree = 0, Neutral = 1, Agree = 2 |
| A6 | Controlling equine endoparasites is beneficial for equine health | ☐ Disagree ☐ Neutral ☐ Agree | Disagree = 0, Neutral = 1, Agree = 2 |
| A7 | Failure to control equine endoparasites can have serious consequences for my animals | ☐ Disagree ☐ Neutral ☐ Agree | Disagree = 0, Neutral = 1, Agree = 2 |

***Note:*** All attitude items are positively worded; no reverse scoring was applied. Attitude scoring: sum of A1-A7 (range 0-14). Categorization based on percentage of maximum score: Poor attitude = 0-7 points (0-50%), Moderate attitude = 8-10 points (57-71%), Good attitude = 11-14 points (79-100%).

**PART D: PRACTICES FOR EQUINE ENDOPARASITE MANAGEMENT (P1-P7)**

**Instructions:** Answer based on what you actually do, not what you think you should do.

| **Item** | **Question** | **Response Options** | **Good Practice (Score = 1)** |
| --- | --- | --- | --- |
| P1 | What type of grazing system do you use for your equines? | ☐ 1. Housekeeping ☐ 2. Free grazing ☐ 3. Semi‑intensive | Housekeeping = 1; Semi‑intensive = 1; |
| P2 | Do you use any method to prevent equine endoparasites? | ☐ 0. No ☐ 1. Yes | Yes = 1 |
| P3 | What is the main method used to prevent equine endoparasites? | ☐ 0. Medication (deworming) ☐ 1. Avoiding contact with other equines ☐ 2. Good management (clean housing, manure removal, pasture hygiene) | Avoiding contact = 1; Good management = 1 |
| P4 | How often do you deworm your equine? | ☐ 0. Never ☐ 1. Occasionally (once a year or less) ☐ 2. Regularly (≥ 2 times per year) | Regularly = 1 |
| P5 | Who decides or administers the deworming? | ☐ 0. Self / traditional decision ☐ 1. Veterinary professional | Veterinary professional = 1 |
| P6 | Do you clean equine housing regularly? | ☐ 0. No ☐ 1. Yes | Yes = 1 |
| P7 | Do you remove manure from housing or grazing areas? | ☐ 0. No ☐ 1. Yes | Yes = 1 |

Practice scoring: Each good practice response scores 1 point; total score = sum of P1-P7 (range 0-7). Categorization based on percentage of maximum score: Poor practice = 0-3 points (0-43%), Moderate practice = 4-5 points (57-71%), Good practice = 6-7 points (86-100%).
