# Supplementary material for: Assessment of Knowledge, Attitudes, and Preventive Practices for Equine Endoparasite Control Among Livestock Owners in South Gondar Zone, Northwest Ethiopia
Source: Vet Med Int. 2026 Jul 22;2026:5535645. doi: 10.1155/vmi/5535645 (PMC13389813; doi:10.1155/vmi/5535645)
Supplement: Supplementary file 2 — Supporting Information 2 Appendix 2: full crosstabs with observed and expected cell counts for all χ²/ FFH exact tests. [file VMI-2026-5535645-s002.docx]

**Appendix 2** Full crosstabs with observed and expected cell counts for all χ²/ Fisher–Freeman–Halton (FFH) Exact tests

| **Outcome** | **Variable** | **Category** | **Outcome Level** | **Observed n** | **Expected n** | **Row %** | **Column %** | **Statistical Test Value** | **P‑value** |
| --- | --- | --- | --- | --- | --- | --- | --- | --- | --- |
| **Knowledge** | Gender | Male | Moderate | 56 | 56.8 | 46.7 | 78.9 | χ² = 0.107, df=1 | 0.744 |
|  |  |  | Good | 64 | 63.2 | 53.3 | 81.0 |  |  |
|  |  | Female | Moderate | 15 | 14.2 | 50.0 | 21.1 |  |  |
|  |  |  | Good | 15 | 15.8 | 50.0 | 19.0 |  |  |
|  | Age | Young | Moderate | 1 | 24.6 | 1.9 | 1.4 | χ² = 65.857, df=2 | < 0.001 |
|  |  |  | Good | 51 | 27.4 | 98.1 | 64.6 |  |  |
|  |  | Adult | Moderate | 54 | 35.9 | 71.1 | 76.1 |  |  |
|  |  |  | Good | 22 | 40.0 | 28.9 | 27.8 |  |  |
|  |  | Old | Moderate | 16 | 10.4 | 72.7 | 22.5 |  |  |
|  |  |  | Good | 6 | 11.6 | 27.3 | 7.6 |  |  |
|  | Education | Illiterate | Moderate | 71 | 35.0 | 95.9 | 100.0 | FFH = 173.213 | < 0.001 |
|  |  |  | Good | 3 | 39.0 | 4.1 | 3.8 |  |  |
|  |  | Primary | Moderate | 0 | 31.2 | 0.0 | 0.0 |  |  |
|  |  |  | Good | 66 | 34.8 | 100.0 | 83.5 |  |  |
|  |  | Secondary | Moderate | 0 | 4.7 | 0.0 | 0.0 |  |  |
|  |  |  | Good | 10 | 5.3 | 100.0 | 12.7 |  |  |
|  | District | Dera | Moderate | 22 | 23.7 | 44.0 | 31.0 | χ² = 0.695, df=2 | 0.706 |
|  |  |  | Good | 28 | 26.3 | 56.0 | 35.4 |  |  |
|  |  | Fogera | Moderate | 26 | 23.7 | 52.0 | 36.6 |  |  |
|  |  |  | Good | 24 | 26.3 | 48.0 | 30.4 |  |  |
|  |  | Libokemkem | Moderate | 23 | 23.7 | 46.0 | 32.4 |  |  |
|  |  |  | Good | 27 | 26.3 | 54.0 | 34.2 |  |  |
|  | Vet Distance | <1 km | Moderate | 4 | 3.8 | 50.0 | 5.6 | FFH = 1.278 | 0.746 |
|  |  |  | Good | 4 | 4.2 | 50.0 | 5.1 |  |  |
|  |  | 1‑5 km | Moderate | 24 | 26.5 | 42.9 | 33.8 |  |  |
|  |  |  | Good | 32 | 29.5 | 57.1 | 40.5 |  |  |
|  |  | 5‑10 km | Moderate | 28 | 27.9 | 47.5 | 39.4 |  |  |
|  |  |  | Good | 31 | 31.1 | 52.5 | 39.2 |  |  |
|  |  | >10 km | Moderate | 15 | 12.8 | 55.6 | 21.1 |  |  |
|  |  |  | Good | 12 | 14.2 | 44.4 | 15.2 |  |  |
| **Attitude** | Gender | Male | Good | 79 | 80.0 | 65.8 | 79.0 | FFH = 1.443 | 0.490 |
|  |  |  | Moderate | 4 | 4.8 | 3.3 | 66.7 |  |  |
|  |  |  | Poor | 37 | 35.2 | 30.8 | 84.1 |  |  |
|  |  | Female | Good | 21 | 20.0 | 70.0 | 21.0 |  |  |
|  |  |  | Moderate | 2 | 1.2 | 6.7 | 33.3 |  |  |
|  |  |  | Poor | 7 | 8.8 | 23.3 | 15.9 |  |  |
|  | Age | Young | Good | 51 | 34.7 | 98.1 | 51.0 | FFH = 41.940 | < 0.001 |
|  |  |  | Moderate | 0 | 2.1 | 0.0 | 0.0 |  |  |
|  |  |  | Poor | 1 | 15.3 | 1.9 | 2.3 |  |  |
|  |  | Adult | Good | 38 | 50.7 | 50.0 | 38.0 |  |  |
|  |  |  | Moderate | 5 | 3.0 | 6.6 | 83.3 |  |  |
|  |  |  | Poor | 33 | 22.3 | 43.4 | 75.0 |  |  |
|  |  | Old | Good | 11 | 14.7 | 50.0 | 11.0 |  |  |
|  |  |  | Moderate | 1 | 0.9 | 4.5 | 16.7 |  |  |
|  |  |  | Poor | 10 | 6.5 | 45.5 | 22.7 |  |  |
|  | Education | Illiterate | Good | 24 | 49.3 | 32.4 | 24.0 | FFH = 87.764 | < 0.001 |
|  |  |  | Moderate | 6 | 3.0 | 8.1 | 100.0 |  |  |
|  |  |  | Poor | 44 | 21.7 | 59.5 | 100.0 |  |  |
|  |  | Primary | Good | 66 | 44.0 | 100.0 | 66.0 |  |  |
|  |  |  | Moderate | 0 | 2.6 | 0.0 | 0.0 |  |  |
|  |  |  | Poor | 0 | 19.4 | 0.0 | 0.0 |  |  |
|  |  | Secondary | Good | 10 | 6.7 | 100.0 | 10.0 |  |  |
|  |  |  | Moderate | 0 | 0.4 | 0.0 | 0.0 |  |  |
|  |  |  | Poor | 0 | 2.9 | 0.0 | 0.0 |  |  |
|  | District | Dera | Good | 31 | 33.3 | 62.0 | 31.0 | FFH = 5.632 | 0.207 |
|  |  |  | Moderate | 4 | 2.0 | 8.0 | 66.7 |  |  |
|  |  |  | Poor | 15 | 14.7 | 30.0 | 34.1 |  |  |
|  |  | Fogera | Good | 31 | 33.3 | 62.0 | 31.0 |  |  |
|  |  |  | Moderate | 2 | 2.0 | 4.0 | 33.3 |  |  |
|  |  |  | Poor | 17 | 14.7 | 34.0 | 38.6 |  |  |
|  |  | Libokemkem | Good | 38 | 33.3 | 76.0 | 38.0 |  |  |
|  |  |  | Moderate | 0 | 2.0 | 0.0 | 0.0 |  |  |
|  |  |  | Poor | 12 | 14.7 | 24.0 | 27.3 |  |  |
|  | Vet Distance | <1 km | Good | 5 | 5.3 | 62.5 | 5.0 | FFH = 4.845 | 0.532 |
|  |  |  | Moderate | 1 | 0.3 | 12.5 | 16.7 |  |  |
|  |  |  | Poor | 2 | 2.3 | 25.0 | 4.5 |  |  |
|  |  | 1‑5 km | Good | 40 | 37.3 | 71.4 | 40.0 |  |  |
|  |  |  | Moderate | 2 | 2.2 | 3.6 | 33.3 |  |  |
|  |  |  | Poor | 14 | 16.4 | 25.0 | 31.8 |  |  |
|  |  | 5‑10 km | Good | 38 | 39.3 | 64.4 | 38.0 |  |  |
|  |  |  | Moderate | 1 | 2.4 | 1.7 | 16.7 |  |  |
|  |  |  | Poor | 20 | 17.3 | 33.9 | 45.5 |  |  |
|  |  | >10 km | Good | 17 | 18.0 | 63.0 | 17.0 |  |  |
|  |  |  | Moderate | 2 | 1.1 | 7.4 | 33.3 |  |  |
|  |  |  | Poor | 8 | 7.9 | 29.6 | 18.2 |  |  |
| **Practice** | Gender | Male | Low | 2 | 3.2 | 1.7 | 50.0 | FFH = 6.031 | 0.050 |
|  |  |  | Moderate | 5 | 7.2 | 4.2 | 55.6 |  |  |
|  |  |  | High | 113 | 109.6 | 94.2 | 82.5 |  |  |
|  |  | Female | Low | 2 | 0.8 | 6.7 | 50.0 |  |  |
|  |  |  | Moderate | 4 | 1.8 | 13.3 | 44.4 |  |  |
|  |  |  | High | 24 | 27.4 | 80.0 | 17.5 |  |  |
|  | Age | Young | Low | 1 | 1.4 | 1.9 | 25.0 | FFH = 44.778 | < 0.001 |
|  |  |  | Moderate | 0 | 3.1 | 0.0 | 0.0 |  |  |
|  |  |  | High | 51 | 47.5 | 98.1 | 37.2 |  |  |
|  |  | Adult | Low | 0 | 2.0 | 0.0 | 0.0 |  |  |
|  |  |  | Moderate | 0 | 4.6 | 0.0 | 0.0 |  |  |
|  |  |  | High | 76 | 69.4 | 100.0 | 55.5 |  |  |
|  |  | Old | Low | 3 | 0.6 | 13.6 | 75.0 |  |  |
|  |  |  | Moderate | 9 | 1.3 | 40.9 | 100.0 |  |  |
|  |  |  | High | 10 | 20.1 | 45.5 | 7.3 |  |  |
|  | Education | Illiterate | Low | 1 | 2.0 | 1.4 | 25.0 | FFH = 5.185 | 0.219 |
|  |  |  | Moderate | 7 | 4.4 | 9.5 | 77.8 |  |  |
|  |  |  | High | 66 | 67.6 | 89.2 | 48.2 |  |  |
|  |  | Primary | Low | 2 | 1.8 | 3.0 | 50.0 |  |  |
|  |  |  | Moderate | 2 | 4.0 | 3.0 | 22.2 |  |  |
|  |  |  | High | 62 | 60.3 | 93.9 | 45.3 |  |  |
|  |  | Secondary | Low | 1 | 0.3 | 10.0 | 25.0 |  |  |
|  |  |  | Moderate | 0 | 0.6 | 0.0 | 0.0 |  |  |
|  |  |  | High | 9 | 9.1 | 90.0 | 6.6 |  |  |
|  | Vet Distance | <1 km | Low | 0 | 0.2 | 0.0 | 0.0 | FFH = 12.243 | 0.021 |
|  |  |  | Moderate | 0 | 0.5 | 0.0 | 0.0 |  |  |
|  |  |  | High | 8 | 7.3 | 100.0 | 5.8 |  |  |
|  |  | 1‑5 km | Low | 0 | 1.5 | 0.0 | 0.0 |  |  |
|  |  |  | Moderate | 1 | 3.4 | 1.8 | 11.1 |  |  |
|  |  |  | High | 55 | 51.1 | 98.2 | 40.1 |  |  |
|  |  | 5‑10 km | Low | 1 | 1.6 | 1.7 | 25.0 |  |  |
|  |  |  | Moderate | 4 | 3.5 | 6.8 | 44.4 |  |  |
|  |  |  | High | 54 | 53.9 | 91.5 | 39.4 |  |  |
|  |  | >10 km | Low | 3 | 0.7 | 11.1 | 75.0 |  |  |
|  |  |  | Moderate | 4 | 1.6 | 14.8 | 44.4 |  |  |
|  |  |  | High | 20 | 24.7 | 74.1 | 14.6 |  |  |
|  | District | Dera | Low | 1 | 1.3 | 2.0 | 25.0 | FFH = 1.492 | 0.836 |
|  |  |  | Moderate | 2 | 3.0 | 4.0 | 22.2 |  |  |
|  |  |  | High | 47 | 45.7 | 94.0 | 34.3 |  |  |
|  |  | Fogera | Low | 2 | 1.3 | 4.0 | 50.0 |  |  |
|  |  |  | Moderate | 4 | 3.0 | 8.0 | 44.4 |  |  |
|  |  |  | High | 44 | 45.7 | 88.0 | 32.1 |  |  |
|  |  | Libokemkem | Low | 1 | 1.3 | 2.0 | 25.0 |  |  |
|  |  |  | Moderate | 3 | 3.0 | 6.0 | 33.3 |  |  |
|  |  |  | High | 46 | 45.7 | 92.0 | 33.6 |  |  |

***Notes:***

- Expected counts = (row total × column total) / 150, rounded to 1 decimal.
- Row % = (observed / row total) × 100; Column % = (observed / column total) × 100.
- For χ² tests, degrees of freedom (df) are shown. For FFH (Fisher‑Freeman‑Halton) tests, the statistic value is shown (e.g., FFH = 173.213).
