# Supplementary material for: Assessment of Knowledge, Attitudes, and Preventive Practices for Equine Endoparasite Control Among Livestock Owners in South Gondar Zone, Northwest Ethiopia
Source: Vet Med Int. 2026 Jul 22;2026:5535645. doi: 10.1155/vmi/5535645 (PMC13389813; doi:10.1155/vmi/5535645)
Supplement: Supplementary file 3 — Supporting Information 3 STROBE‐R3 [file VMI-2026-5535645-s003.docx]

**STROBE**

| **Main Section** | **Item No** | **Recommendation** | **Reported on Page Number** |
| --- | --- | --- | --- |
| **Title and abstract** | 1 | (a) Indicate study design in title or abstract | 1 (Title: “Assessment of Knowledge, Attitudes, and Preventive Practices for Equine Endoparasite Control among Livestock Owners in South Gondar Zone, Northwest Ethiopia: A Community‑Based Cross‑Sectional Study”); 2 (Abstract: “A community‑based cross‑sectional study was conducted”) |
|  |  | (b) Provide informative summary of what was done and found | 2 (Full abstract – single paragraph) |
| **Introduction** |  |  |  |
| Background/rationale | 2 | Explain scientific background and rationale | 3‑4 (Paragraphs 1‑3) |
| Objectives | 3 | State specific objectives, including any prespecified hypotheses | 4 (Final paragraph lists four specific objectives) |
| **Methods** |  |  |  |
| Study design | 4 | Present key elements of study design early | 5 (Section 2.2: “A community‑based cross‑sectional study design was employed”) |
| Setting | 5 | Describe setting, locations, and relevant dates | 5 (Section 2.1: study area); 5 (Section 2.2: “September 2023 to July 2024”) |
| Participants | 6 | (a) Give eligibility criteria and selection methods | 6 (Section 2.3: adult responsible owners); 6‑7 (Section 2.5: multistage sampling) |
| Variables | 7 | Clearly define outcomes, exposures, predictors, confounders | 6 (Section 2.4: categorization); 7‑8 (Section 2.7: operational definitions of KAP outcomes) |
| Data sources/measurement | 8 | For each variable, give sources of data and details of assessment | 7 (Section 2.6: questionnaire, pre‑testing, trained technicians); Supplementary File S1 |
| Bias | 9 | Describe efforts to address potential bias | 7 (Section 2.6: pre‑testing, training, daily checking) |
| Study size | 10 | Explain how study size was arrived at | 6 (Section 2.5: formula n = 0.25/(SE)² → n=150) |
| Quantitative variables | 11 | Explain handling of quantitative variables, groupings | 6 (Section 2.4: age groups young<30, adult30‑45, old>45) |
| Statistical methods | 12 | (a) Describe all statistical methods, including confounder control | 8‑9 (Section 2.8: χ², FFH, univariable and multivariable binary logistic regression, backward stepwise) |
|  |  | (b) Describe methods for subgroup and interaction analyses | 8‑9 (Section 2.8: subgroup analyses by gender, age, education, district, distance) |
|  |  | (c) Explain handling of missing data | 8 (Section 2.7: daily completeness checks – no missing data) |
|  |  | (d) Describe analytical methods accounting for sampling strategy | 6 (Section 2.5: multistage sampling); 8‑9 (Section 2.8: analyses account for design) |
|  |  | (e) Describe any sensitivity analyses | Not applicable (none performed) |
| **Results** |  |  |  |
| Participants | 13 | (a) Report numbers at each stage of study | 9 (Section 3.1: “150 questionnaires completed”); Fig. 2 |
|  |  | (b) Give reasons for non‑participation | None – all selected participated |
|  |  | (c) Consider use of a flow diagram | Fig. 2 |
| Descriptive data | 14 | (a) Give characteristics of study participants | 9 (Section 3.1 narrative); Table 1 |
|  |  | (b) Indicate number with missing data for each variable | Table 1 (no missing data; all n=150) |
| Outcome data | 15 | Report numbers of outcome events or summary measures | 9‑10 (Sections 3.2‑3.4); Tables 2,3,4 |
| Main results | 16 | (a) Give unadjusted and adjusted estimates with precision | 12‑14 (Tables 8,9,10: COR and AOR with 95% CI, p‑values; footnotes indicate confounders adjusted) |
|  |  | (b) Report category boundaries for continuous variables | 6 (Section 2.4: age); 8 (Section 2.7: KAP score categories 0‑50%, 51‑74%, 75‑100%) |
|  |  | (c) Translate relative risk into absolute risk if relevant | Not applicable |
| Other analyses | 17 | Report other analyses (subgroups, interactions, sensitivity) | 9‑10 (Tables 2‑4 bivariate); 12‑14 (Tables 8‑10 multivariable) |
| **Discussion** |  |  |  |
| Key results | 18 | Summarize key results with reference to objectives | 16 (Section 4.1: overall KAP levels 52.7%, 66.7%, 91.3%) |
| Limitations | 19 | Discuss limitations, potential bias, imprecision | 27 (Section 4.7: cross‑sectional, self‑report bias, sparse data/wide CIs, limited generalizability, no parasitological validation; questionnaire provided as Supplementary File S1) |
| Interpretation | 20 | Give cautious overall interpretation considering limitations and similar studies | 16‑25 (Sections 4.1‑4.5: comparisons with Yizengaw et al., 2026; Seyoum et al., 2017; Shrubb et al., 2025; Merlin & Delerue, 2025) |
| Generalizability | 21 | Discuss external validity of study results | 25 (Section 4.5: applicability to similar rural settings) |
| **Other information** |  |  |  |
| Funding | 22 | Give source of funding and role of funders | 28 (Funding section: “This work did not receive any grant…”) |
